# Heart Rate Variability Biofeedback for Mild Traumatic Brain Injury: A Randomized-Controlled Study

**DOI:** 10.1007/s10484-023-09592-4

**Published:** 2023-06-19

**Authors:** Hsueh Chen Lu, Richard Gevirtz, Chi Cheng Yang, Alexander O. Hauson

**Affiliations:** 1 California School of Professional Psychology at Alliant International University, Clinical Psychology PhD Program, San Diego, CA USA; 2 Department of Psychiatry, University of California San Diego, La Jolla, San Diego, CA USA; 3 Institute of Brain Research and Integrated Neuropsychological Services (iBRAINS.Org), San Diego, CA USA; 4 Department of Psychology, National Chengchi University, Taipei, Taiwan

**Keywords:** Mild traumatic brain injury, Heart rate variety biofeedback, Central and autonomic nervous systems, Neuropsychological functioning, Heart rate variability, Rehabilitation

## Abstract

To determine whether heart rate variability biofeedback (HRV-BF) training, compared to a psychoeducation control condition can strengthen the integration of the central and autonomic nervous systems as measured by neuropsychological measures in patients with mild traumatic brain injury (mTBI). Participants were recruited from two university hospitals in Taipei, Taiwan. A total of 49 participants with mTBI were recruited for this study. Forty-one participants completed the study, 21 in the psychoeducation group and 20 in the HRV-BF group. Randomized controlled study. The Taiwanese Frontal Assessment Battery, the Semantic Association of Verbal Fluency Test, the Taiwanese version of the Word Sequence Learning Test, the Paced Auditory Serial Addition Test-Revised, and the Trail Making Test were used as performance-based neuropsychological functioning measures. The Checklist of Post-concussion Symptoms, the Taiwanese version of the Dysexecutive Questionnaire, the Beck Anxiety Inventory, the Beck Depression Inventory, and the National Taiwan University Irritability Scale were used as self-report neuropsychological functioning measures. Furthermore, heart rate variability pre- vs. post-training was used to measure autonomic nervous system functioning. Executive, information processing, verbal memory, emotional neuropsychological functioning, and heart rate variability (HRV) were improved significantly in the HRV-BF group at the posttest whereas the psychoeducation group showed no change. HRV biofeedback is a feasible technique following mild TBI that can improve neuropsychological and autonomic nervous system functioning. HRV-BF may be clinically feasible for the rehabilitation of patients with mTBI.

**Supplementary Information:**

The online version contains supplementary material available at 10.1007/s10484-023-09592-4.

## Introduction

The term traumatic brain injury (TBI) refers to a direct or indirect force that causes an injury to the brain, and this injury can occur as a result of direct blows to the head, face, neck, or any other location on the body accompanied by an impulsive force (Lezak et al., 2004; McCrory et al., 2017). Mild traumatic brain injury (mTBI) is defined as an injury with a Glassgow Coma Scale score (GCS) of 13–15, with loss of consciousness less than 30 min, and with post traumatic amnesia (PTA) of less than 24 h (American Congress of Rehabilitation Medicine, 1993). Patients with mTBI frequently remit or recover within hours or 1 or 2 months, but some patients may live with symptoms for years (Alexander, 1995; Bazarian et al., 2005; Frencham et al., 2005; McCrory et al., 2017; Schretlen & Shapiro, 2003). Around 80% of traumatic brain injuries are mild, and approximately 15%-25% are related to post-concussion syndrome (PCS) (Bazarian et al., 2005; Konrad et al., 2011; Ruff & Weyer Jamora, 2009; Willer & Leddy, 2006). It is important to note that the acute clinical signs and symptoms are generally the result of a functional impairment rather than a structural injury, and thus structural neuroimaging reveals no abnormalities (McCrory et al., 2017).

Brain injury results in central nervous system damage and also impacts the peripheral nervous system (Blennow et al., 2012; Hayes et al., 2016; Johnson et al., 2013; Jünger et al., 1997; Strebel et al., 1997). Increased arousal of the sympathetic nervous system and underactivation of the parasympathetic nervous system are correlated with mTBI post-injury and with post-concussion syndrome (Lagos etal., 2013). Heart rate variability (HRV) can be an indicator of central-peripheral neural feedback and the integration of the central nervous and autonomic nervous systems (Thayer & Brosschot, 2005; Thayer et al., 2009). Decreased HRV has been found in patients with mTBI (La Fountaine et al., 2009). Attention, memory, and executive functioning deficits are common outcomes for all severities of TBI (Arciniegas et al., 2002). Patients who have acute mTBI less than three months post injury experience the greatest impacts in delayed memory, working memory, fluency, processing speed, attention, and executive functioning (Belanger & Vanderploeg, 2005; Belanger et al., 2005; Kwok et al., 2008; McAllister et al., 2006; Willmott et al., 2009; Wozniak et al., 2007).

One intervention that targets autonomic function is vagal nerve stimulation (VNS) (Mondal et al., 2019; Neren et al., 2016; Stefan et al., 2012). The vagus nerve, the tenth cranial nerves, consists of both afferent and efferent fibers that connect the brain and internal organs, with afferent fibers making up more than 80% of the total fibers (Srihagulang et al., 2022). The stimulation of the vagal nerve has been found to significantly affect brain activity as well as diffusely projecting nuclei of the brainstem such as the locus coeruleus, the nucleus of the solitary tract (NST), the thalamus, as well as limbic structures (Cavanna & Bagary, 2011). The brainstem nucleus of the solitary tract (NST) serves as the primary relay station for afferent vagal nerve fibers, and it has wide projections to numerous regions of the forebrain, brainstem, the thalamus, as well as areas associated with learning and memory formation, such as the amygdala and the hippocampal region (Cavanna & Bagary, 2011; Groves & Brown, 2005). Several cognitive effects of VNS have been found to be beneficial, including the ability to improve working memory as well as emotional reactivity and regulation in the clinical population (Sun et al., 2017). The therapeutic effects of VNS have been studied in both animal and human studies of TBI. Researchers have found that VNS improves cognitive and motor functions in rats with TBI, as well as reducing secondary brain injury (Smith et al., 2005, 2006; Srihagulang et al., 2022). In human studies, VNS also improved consciousness in patients suffering from vegetative state/unresponsive wakefulness syndrome following severe traumatic brain injury (Noé et al., 2020). As of yet, researchers have not found any studies demonstrating that VNS is effective in improving cognitive function in patients who suffer from brain injuries.

In a manner similar to VNS, based on the heart-brain integration in HRV, the intervention of HRV-BF can validate the neurovisceral relationship and improve the interaction between top-down and bottom-up processes. HRV-BF also referred to as resonance frequency (RFF) biofeedback applies heart rate variation that accompany breathing, and diaphragmatic breathing to stimulate afferent and efferent vagal pathways to influence brain areas (locus coeruleus, orbitofrontal cortex, insula, hippocampus, and amygdala) (Lehrer et al., 2000; Lehrer & Gevirtz, 2014; Grundy, 2002; Del Pozo et al., 2004). In addition, HRV-BF interventions have been shown to enhance cognitive abilities (attention, working memory, short-term memory, inhibition) in non-clinical and clinical populations (Ginsberg et al., 2010; Jester et al., 2019; Pop-Jordanova & Chakalaroska, 2008; Prinsloo et al., 2011).

Few previous studies have addressed the treatment outcomes of HRV-BF in TBI, and such studies were mostly limited to pilot studies or case reports. For example, a case study with a 42-year-old athlete who sustained a concussion and severe post-concussion syndrome (Lagos et al., 2013). After ten weeks of HRV-BF training, the patient showed a significant increase in HRV and improvement in cardiovagal activity, as well as a reduction in mood disturbance, post-concussion symptoms, and headache severity (Lagos et al., 2013). A study by Kim and her colleagues focused on thirteen individuals with various types of severe brain injury (e.g., TBI, anoxia, or an aneurysm) who were on average twenty-four years post-injury (Kim et al., 2015). After ten sessions of HRV-BF training, participants showed a significant increase in HRV, and HRV was associated with improved sustained attention and emotional control (Kim et al., 2015). However, Francis and her colleagues showed that thirty individuals with severe TBI in a treatment group and thirty individuals in a control group had no significant differences in resting HRV after a single session of HRV-BF (Francis et al., 2016). In a study that compared twenty-five individuals with severe TBI given an HRV-BF intervention to twenty-five individuals in the waitlist group, Wearne and his colleagues reported a greater reduction in depression and sleep disturbances for the HRV-BF group (Wearne et al., 2021). To date, however, few studies have examined the effectiveness of HRV-BF in treating brain injury, and only one case study has examined the effects of HRV-BF intervention for mild traumatic brain injury (Lagos et al., 2013).

Several studies have demonstrated the effectiveness of psychoeducational interventions in treating mTBI as well as supplementing mental health disorders in the acute and chronic phases (Comper et al., 2005; Jones et al., 2020). There is strong empirical support for psychoeducational early interventions after mild traumatic brain injury, with several systematic reviews indicating that they are effective (Caplain et al., 2019; Comper et al., 2005; Cooper et al., 2015; Snell et al., 2009). Psychoeducation typically has significant advantages over cognitive rehabilitation, such as early intervention and short intervention times. In this study, we aim to determine whether HRV-BF training, compared to a psychoeducation group, can strengthen the integration of the central and autonomic nervous systems and improve neurophysiological function in patients with mTBI. Thus, it is hypothesized that participants receiving the HRV-BF intervention will demonstrate greater improvements in neuropsychological (executive functions, information processing, verbal memory, and psychological symptoms) and physiological functions (HRV indices and post-concussion symptoms) compared to the psychoeducation condition between pre- and post-treatment tests.

## Methods

### Study Design

**Fig. 1:**
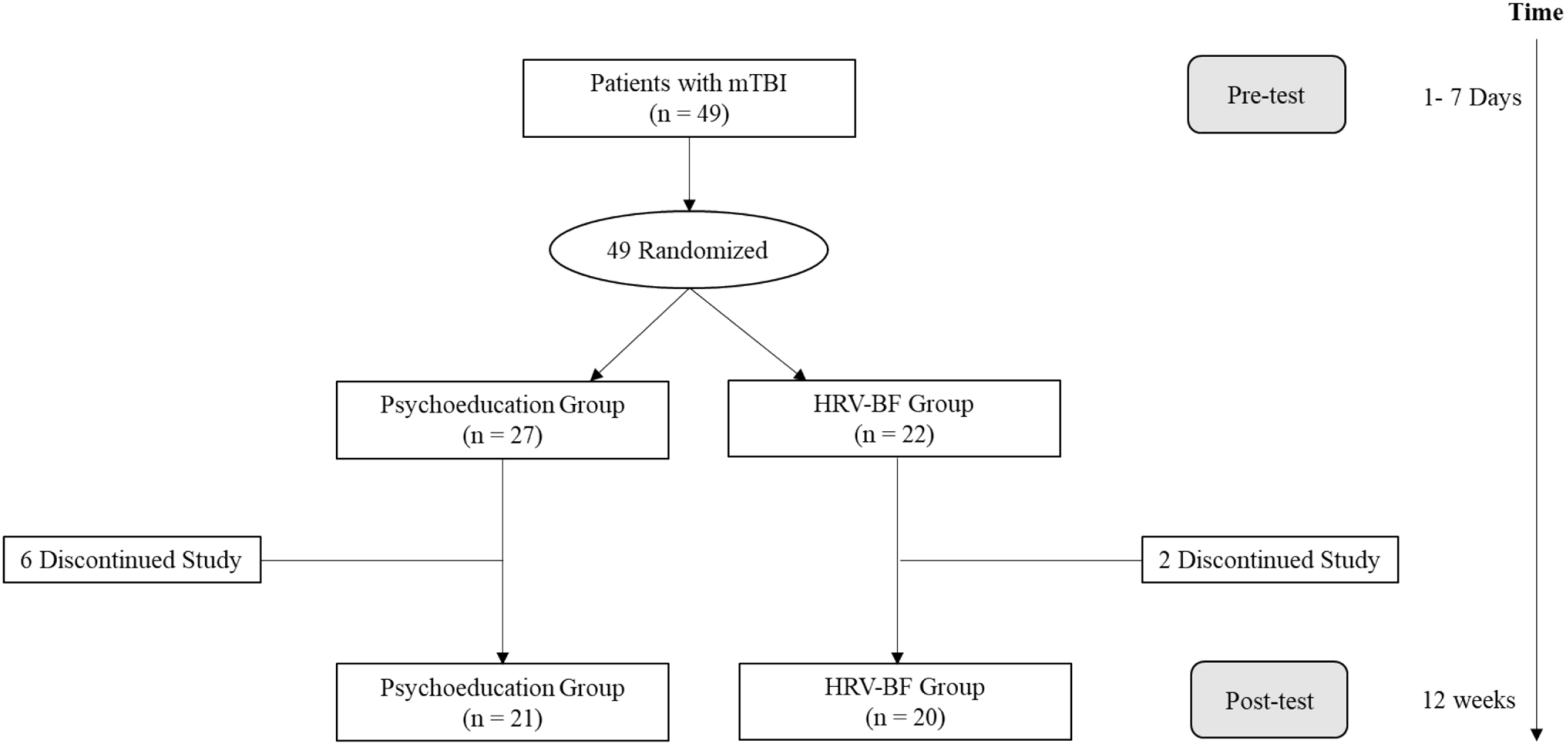
Flow chart of participants through the study

The study protocol was approved by the Ethics Committees of the Alliant International University (IRB number: 1908147259), the National Taiwan University Hospital (IRB number: 201204004RIC), and the Fu Jen Catholic University Hospital (IRB number: FJUH109016). A randomized controlled trial design was adopted so that participants were randomly assigned to either the psychoeducation group or the HRV-BF intervention group before the intervention. Randomization was performed by assigning random numbers from random number tables to the psychoeducation and HRV-BF interventions. The psychoeducation group received standard medical care and one 60-min session of post-brain injury psychoeducation and post-test measurement. The HRV-BF group received standard medical care and a weekly 60-min session of HRV-BF intervention for ten weeks and then participated in a post-test measurement (Fig. 1).

### Participants

Patients were referred from National Taiwan University Hospital and Fu Jen Catholic University Hospital. They were referred from the emergency department and diagnosed with mTBI by neurosurgeons in an outpatient program. The diagnosis of mTBI was based primarily on the World Federation of Neurosurgical Societies criteria (Servadei et al., 2001; Shukla & Devi, 2010): (a) a Glasgow Coma Scale score of 14 or15 and (b) the presence or absence of one or more of the neurological symptoms, including loss of consciousness, posttraumatic amnesia, vomiting, or diffuse headache. Accordingly, the inclusion criteria were that patients were between 18 and 65 years of age and sustained a brain injury within 7 days. Patients were excluded if they reported (a) psychiatric illness or other medical conditions (cerebrovascular, brain tumor, cancer) and/or (b) an inability to speak or understand Mandarin fluently. A total of 49 participants were recruited for the study, and 5 participants dropped out of the psychoeducation group, and 1 participant dropped out of the HRV-BF group after the pre-test due to losing contact or no longer wishing to participate. At the post-test, 1 participant from the psychoeducation group dropped out of the study due to transfer to another hospital, and 1 participant from the HRV-BF group discontinued at the end of the fourth session due to being unable to schedule and complete the rest of the interventions (Fig. 1).

### Interventions

Participants randomized to the psychoeducation group received a 60-min psychoeducation session both orally and on paper and were given a booklet to take home. The psychoeducation included standardized information about the causes, incidence, and possible consequences of mTBI, as well as advice on strategies for post-brain injury symptom reductions, memory and attention improvement, stress and emotional regulation, and sleep hygiene (Table 1). Participants randomized to the HRV-BF group received a 10-week HRV-BF protocol. The HRV-BF protocol adopted the protocol of Lehrer et al. (2000), which included diaphragmatic breathing, paced breathing, and RSA biofeedback. Briefly, in the first session, we set up the pacing stimulus and instructed participants to breathe at five different frequencies (6.5, 6.0, 5.5, 5.0, and 4.5 cycles/min, respectively) for two minutes to determine the individual best resonance frequency by finding the frequency peak at 0.1 Hz. In subsequent sessions, the goal of HRV-BF was to maximize LF power at 0.1 Hz and RSA by using the cardio tachometer display as biofeedback. In addition, we provided a KYTO Bluetooth mobile heart rate monitor with an ear clip and used the smartphone app technology Elite HRV for HRV-BF home exercise sessions. Participants were assigned two 20-min home exercise sessions daily from the first session (Table 2).

**Table 1:** The Psychoeducation Session Component

Psychoeducation
Session 1 - General exploration of mTBI: typical symptoms, deficits, and time course - Understanding post-concussion syndrome: (1) Physical symptoms (2) Cognitive symptoms (3) Emotional symptoms
- Sleep hygiene - Stress reduction strategies: (1) Problem-solving orientation (2) Emotionally focused orientation
- Strategies for coping with physical symptoms: (1) Adjusting workload and achieving pre-injury performance gradually (2) Rest adequately and avoid overloading
- Strategies for coping with cognitive symptoms: (1) Concentration and memory: be inquisitive, reduce distractions, and use compensatory strategies
- Strategies for coping with emotional symptoms (depression, anxiety, anger**)**: (1) Examine thoughts and check facts (2) Behavioral activation (3) Stress and anger management (4) Maintain friends and family support networks

**Table 2:** The Heart Rate Variability Biofeedback Session Component

HRV-BF
Session 1 - An introduction to the HRV-BF - Estimating the resonance frequency for the individual - Providing abdominal breathing training instructions that emphasize slow, stable, and smooth breathing - Homework: the participant was instructed to practice breathing comfortably at his/her resonance frequency by connecting the KYTO Bluetooth mobile heart rate monitor to the ear clip, as well as using the Elite HRV smartphone app as a tool for HRV-BF home exercise by exhaling more than inhaling for two 20-min periods
Session 2 - Reviewing home practice records through the Elite HRV dashboard (HRV tracking, identifying trends and behavior patterns, and detailed reporting)
Session 3 - Reviewing home practice records through the Elite HRV dashboard (HRV tracking, identifying trends and behavior patterns, and detailed reporting) - A review of abdominal breathing with slow exhalations at the resonance frequency - A cardiotachometer display is used as biofeedback to instruct the participant to maximize RSA. Breathing in phase with HR changes and maximizing heart rate changes is instructed to the participant - Homework: the participant was instructed to practice breathing comfortably at his/her resonant frequency by connecting the KYTO Bluetooth mobile heart rate monitor to the ear clip, as well as using the Elite HRV smartphone app as a tool for HRV-BF home exercise by exhaling more than inhaling for two 20-min periods
Session 4–9 - Reviewing home practice records through the Elite HRV dashboard (HRV tracking, identifying trends and behavior patterns, and detailed reporting) - A cardiotachometer display is used as biofeedback to instruct the participant to maximize RSA. Breathing in phase with HR changes and maximizing heart rate changes is instructed to the participant - Homework: the participant was instructed to practice breathing comfortably at his/her resonant frequency by connecting the KYTO Bluetooth mobile heart rate monitor to the ear clip, as well as using the Elite HRV smartphone app as a tool for HRV-BF home exercise by exhaling more than inhaling for two 20-min periods
Session 10 - Reviewing home practice records through the Elite HRV dashboard (HRV tracking, identifying trends and behavior patterns, and detailed reporting) - A cardiotachometer display is used as biofeedback to instruct the participant to maximize RSA. Breathing in phase with HR changes and maximizing heart rate changes is instructed to the participant - Review the treatment effectiveness of HRV-BF

### Outcome Measures

Primary outcomes included measures of executive functioning, information processing, verbal memory, and emotional neuropsychological functioning (performance-based and self-reported neuropsychological measures), and the autonomic nervous system function. Demographic characteristics and medical conditions were collected from the patient’s demographic forms and medical records.

### Performance-based Neuropsychological Functioning Measure

#### Taiwanese Frontal Assessment Battery (TFAB)

The TFAB was used to assess overall executive function and comprises six subtests (conceptualization, mental flexibility, motor programming, sensitivity to interference, inhibitory control, and environmental autonomy) to assess specific domains of executive function (Wang et al., 2016). The measure has fair to good psychometric properties such as Cronbach’s alpha of 0.70 and test–retest reliability: *r* = 0.88. Scores on each subtest range from 0 to 3, resulting in an overall TFAB score of 18 (Wang et al., 2016).

#### The Semantic Association of Verbal Fluency Test (SVF)

As a measure of executive function, the SVF test assessed word productivity and semantic fluency (Hua et al., 1997; Troyer, 2000; Wang et al., 2016). The SVF test examined three semantic categories: Fruit, Fish, and Vegetable. Participants were given a time limit of one minute to report as many items as possible within a given category. The score was calculated by adding the correct answers from each of the three categories.

#### The Taiwanese Version of the Word Sequence Learning Test (TWSLT)

The TWSLT was used to assess verbal memory function (Hamsher et al., 1983; Hua, 1986). A 10-trial presentation of six Chinese semantically meaningless words is used to test learning and recall abilities. Participant’s performance was assessed using immediate recall, 10-min delayed free recall, cued recall, and recognition tasks.

#### The Paced Auditory Serial Addition Test-Revised (PASAT-R)

The PASAT-R was used to assess the ability to process auditory information (Lezak et al., 2004). It consists of four trials, each of which presented 26 randomly digits (1–9), and a total of 100 responses were recorded. Participants were instructed to add each digit to the one immediately preceding it. For example, when the digits ‘2–1-3’ were given, the correct responses beginning with ‘1’ were ‘3–4’. The speed of the digital presentations varied by 0.4 s between 1.6 and 2.8 s.

#### The Trail Making Test (TMT)

The TMT was to assess abilities of scanning, visuomotor tracking, and cognitive flexibility. Participants were presented with two worksheets. On Worksheet A, participants were asked to draw lines connecting consecutively numbered circles, while on Worksheet B, they were asked to connect consecutively numbered circles and circles with lettered by alternating sequences of numbers and letters. The TMT score is determined by the reaction time (seconds) required for each worksheet (Reitan, 1958).

### Self-reported Neuropsychological Functioning Measure

#### Checklist of Post-concussion Symptoms (CPCS)

The CPCS was used to assess post-concussion symptoms. It is based on 16 common post-concussion symptoms after a mTBI as defined by the International Classification of Diseases (Yang et al., 2007). CPCS is self-rated on a five-point Likert scale. A score of ‘0’ means no symptoms are present, ‘1’ means mild symptoms are present, ‘2’ means moderate symptoms are present, and a score of ‘3’ means severe symptoms are present.

#### Taiwanese Version of Dysexecutive Questionnaire (TDEX)

The TDEX was used to assess dysexecutive function in daily life (Hu & Yang, 2015). A five-point Likert scale (0 = ‘never’, 4 = ‘very often’) was used to assess the frequency of dysexecutive behaviors reported on the TDEX, with a higher score indicating a higher frequency.

#### Beck Anxiety Inventory (BAI)

The Chinese version of the BAI, which consists of 21 items on a four-point Likert scale, was used to assess anxiety symptoms. The Chinese version of BAI has good reliability and validity (e.g., Cronbach's α = 0.85; test–retest reliability: *r* = 0.75) (Lin, 2000). Participants were asked to rate their anxiety symptoms over the past week; the higher the BAI scores, the more severe the symptoms.

#### Beck Depression Inventory (BDI)

The Chinese version of the Beck Depression Inventory—2nd edition (BDI-II), which consists of 21 items and is rated on a four-point Likert scale (Chen, 2000). BDI-II has good psychometric properties, such as high internal consistency (Cronbach’s r = 0.93) and test–retest reliability (r = 0.93). The BDI-II measured depression symptoms over the past two weeks, with a higher score indicating more severe symptoms.

#### The National Taiwan University Irritability Scale (NTUIS)

The National Taiwan University Irritability Scale was developed to assess irritability after a head injury (Yang et al., 2011). The NTUIS is an 18-item, six-point Likert scale that measures annoyance and verbal aggression. Items were rated on a scale from 1 (does not correspond to the participant's condition) to 6 (perfectly corresponds to the participant's condition).

### Psychophysiological Measure- Autonomic Nervous System Function

Participants in both conditions underwent assessment of their ANS function during study Week 1 (pre-test) and Week 12 (post-test). All physiological measurements were performed with non-invasive electrodes using PhysioPilot GP-8e hardware and PhysioPilot software for Windows. Heart rate was measured with electrodes placed under both the right and left clavicles and under the left lower ribs. A breathing belt was used to monitor respiratory rate. A 10-min baseline (quiet sitting) was used to measure autonomic function.

## Data Analysis

### Electrocardiogram (ECG) Data Processing

For analysis of physiological data collected during pre-and post-tests, HRV data were collected and exported in Inter-Beat Interval (IBI) format to Kubios HRV Standard 3.3.1 software for Windows. Visual inspection for premature ventricular contractions, arrhythmia, or movement ECG artifacts and a medium-strength artifact correction algorithm was used to adjust and clean the physiological data with the Kubios software. ECG data were excluded if they contained more than 5% artifacts (Laborde et al., 2017). IBI was transformed into the time domain and frequency domain of HRV. The time domain of HRV was measured using the standard deviation of normal-to-normal intervals (SDNN) to reflect total HRV. To determine vagal activity, the root mean square of successive differences (RMSSD) and the natural logarithm of high frequency (lnHF) were used to measure HRV in the time and frequency domains (Shaffer & Ginsberg, 2017; Shaffer et al., 2014).

### Statistical Analysis

Statistical analysis was performed using the software program IBM Statistical Package for Social Sciences (SPSS) 27 for Windows. A comparison of demographic data and research-related variables at the pretest between the psychoeducation and HRV-BF groups was performed with F tests and chi-square tests to determine the equivalence of the groups. For neuropsychological and physiological measures, two-factor ANOVAs were used to analyze the interaction effect of groups (psychoeducation and HRV-BF groups) x time (pretest and posttest). The effect size was determined by partial eta squared (*η*^*2*^_*p*_) and cohen's d (d), with a small effect size considered *η*^*2*^_*p*_ < 0.058 and d ≤ 0.20, a medium effect size considered *η*^*2*^_*p*_ between 0.058 and 0.138, and d between 0.50 and 0.80, and *η*^*2*^_*p*_ > 0.138 and d ≥ 0.80 indicates a large effect (Cohen, 1988).

## Results

### Participant Characteristics and the Group Equivalence at Pretest

A total of 49 patients with mTBI were recruited for this study. Forty-one of these participants completed the study (Fig. 1). Group equivalence was assessed based on demographic and outcome measures for each group at pretest. The two groups did not differ significantly in demographic characteristics, including age, gender, occupation, marital status, years of education, pre-existing medical conditions, or health behaviors (Table 3). In terms of injury characteristics, there were no significant differences between the psychoeducation and HRV-BF groups in the number of pre-head injuries, the interval of head injury to pre-test, post-traumatic amnesia, or cause of head injury. Also, a Chi-square test indicated that the number of participants who completed the study did not differ significantly between the groups, *χ*^*2*^ (1) = 1.53, *p* = 0.216. However, there was a significant difference in the number of people experiencing loss of consciousness (LOC) after the head injury, *χ*^*2*^ (1) = 7.04, *p* = 0.008, such that more HRV-BF group participants experienced loss of consciousness after the head injury than the psychoeducation group participants (Table 3). Similarly, one-way ANOVAs did not reveal any significant pre-treatment differences between the psychoeducation and HRV-BF groups on the neuropsychological and physiological measures (Tables 4 and 5).

**Table 3:** Group Equivalence on Pre-Treatment Demographic Characteristics

Variable	Psychoeducation (n = 21)	HRV-BF (n = 20)	*F*/ *χ*^*2*^	*P*
Age (years)	34.29 (14.00)	40.05 (14.22)	*F* = 1.71	.199
Gender (n)				
Male	15	11	*χ*^*2*^ = 1.19	.275
Female	6	9		
Occupation (n)				
No occupation	4	3	*χ*^*2*^ = 7.92	.339
Professional	2	3		
Technicians/ associate professionals	1	3		
Clerical support workers	0	3		
Services/ sales workers	9	6		
Craft/ related trades workers	2	2		
Plant/ machine operators/ assemblers	1	0		
Elementary occupations	2	0		
Marital status (n)				
Single/ unmarriage	13	8	*χ*^*2*^ = 2.64	.267
Marriage/ living together	8	11		
Divorce/ not living together	0	1		
Years of education	13.90 (2.12)	13.15 (2.98)	*F* = .88	.354
Injury characteristics				
The number of pre-head injuries (n)				
Non	13	11	*χ*^*2*^ = 1.39	.498
One time	7	9		
Over two times	1	0		
The interval of head injury to pre-test (days)	3.62 (2.36)	3.05 (1.76)	*F* = .76	.388
Loss of consciousness (n)				
No	15	6	*χ*^*2*^ = 7.04**	.008
Yes	6	14		
Post-traumatic amnesia (n)				
No	15	9	*χ*^*2*^ = 2.95	.086
Yes	6	11		
Cause of head injury (n)				
Transportation Accident	12	12	*χ*^*2*^ = 1.23	.746
Fall	5	4		
Hit	3	4		
Assault	1	0		
Pre-existing medical conditions				
Cardiovascular diseases (n)				
No	20	17	*χ*^*2*^ = 1.22	.269
Yes	1	3		
Health behaviors				
Exercises (n)				
No	10	10	*χ*^*2*^ = .02	.879
Yes	11	10		
Yoga, qigong, breathing exercises (n)				
No	19	16	*χ*^*2*^ = .90	.343
Yes	2	4		

*P < .05, **P < .01, *** *P* < .001

**Table 4:** Group Equivalence on Neuropsychological Measures at Baseline

Variable	Psychoeducation (n = 21)	HRV-BF (n = 20)	*F*	*P*
Executive Function				
TFAB	14.29 (2.33)	13.65 (2.18)	.81	.373
SVF	37.29 (9.14)	33.10 (8.17)	2.38	.131
TMT-B	82.87 (53.74)	83.33 (36.97)	.00	.975
TDEX	22.38 (12.03)	23.60 (11.39)	.11	.741
Information Processing				
PASAT	72.24 (21.18)	74.60 (14.94)	.17	.684
TMT-A	36.21 (11.30)	39.51 (15.59)	.61	.441
Verbal Memory				
Verbal learning				
Immediate recall	52.90 (6.35)	51.30 (6.84)	.61	.441
Delayed free recall	2.19 (1.54)	2.20 (1.58)	.00	.984
Cued recall	3.33 (1.59)	3.30 (1.81)	.00	.950
Recognition	26.67 (3.15)	26.95 (2.44)	.10	.061
Symptom / Emotional Function				
CPCS	12.19 (8.81)	15.40 (10.52)	1.13	.295
BAI	6.90 (6.64)	8.55 (6.85)	.61	.439
BDI	9.00 (6.57)	7.75 (8.30)	.29	.595
NTUIS	49.38 (20.55)	49.85 (12.79)	.01	.931

*P < .05, **P < .01, *** *P* < .001

**Table 5:** Group Equivalence on Physiological Measures at Baseline

Variable	Psychoeducation (n = 21)	HRV-BF (n = 20)	*F*	*P*
HR (bpm)	80.24 (10.84)	77.15 (11.09)	.81	.373
SDNN (ms)	22.74 (11.61)	21.09 (8.65)	.27	.610
RMSSD (ms)	19.79 (11.95)	19.06 (7.81)	.05	.819
lnHF (ms^2^)	4.87 (1.40)	4.70 (1.15)	.18	.672
lnLF (ms^2^)	5.28 (1.10)	5.12 (1.02)	.24	.625
LF/HF (%)	2.13 (1.80)	2.53 (2.74)	.31	.584

*P < .05, **P < .01, *** *P* < .001

### Main Analysis of Outcome Measure

**Fig. 2:**
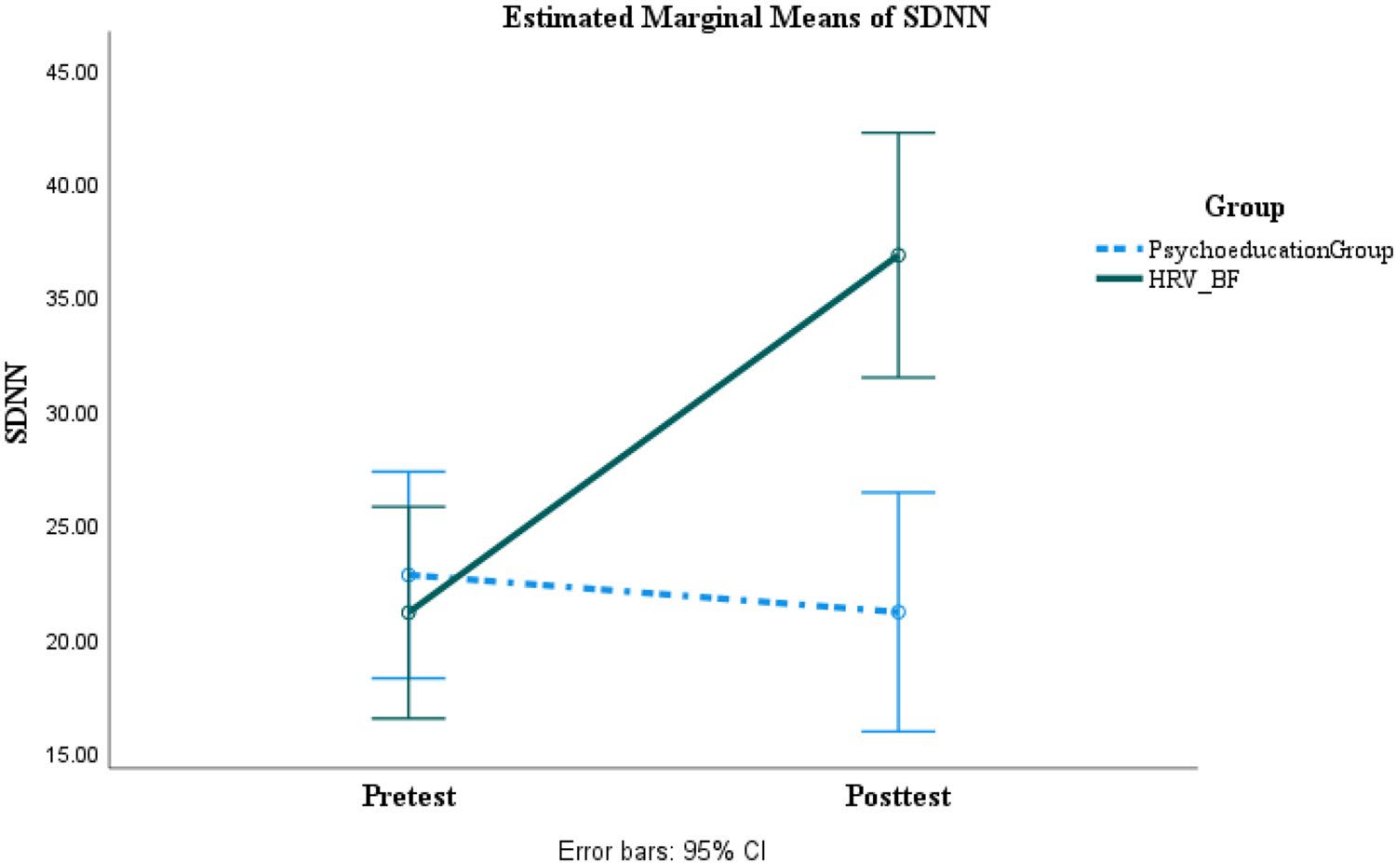
SDNN changes across time between the psychoeducation and the HRV-BF groups

**Fig. 3:**
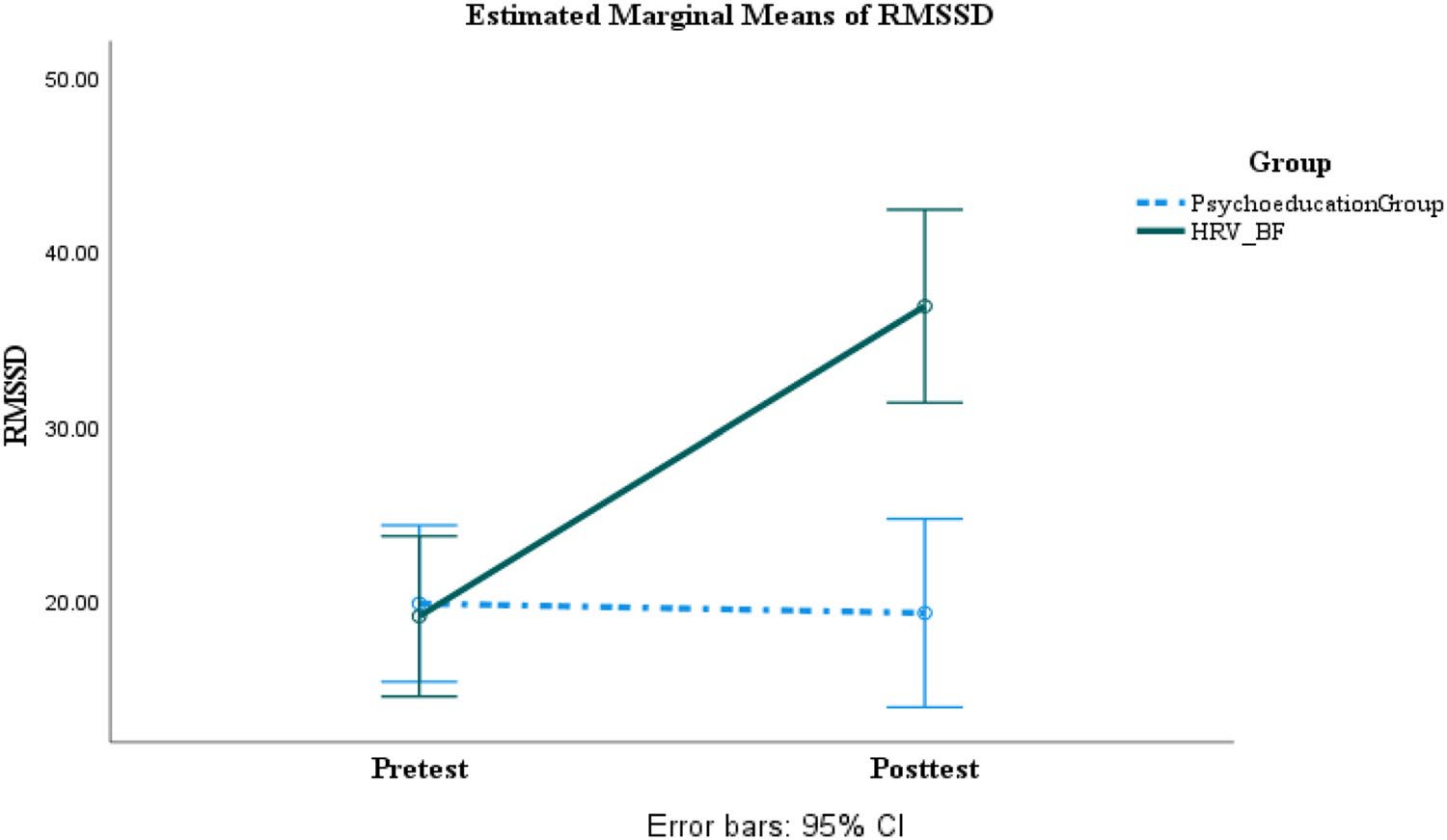
RMSSD changes across time between the psychoeducation and the HRV-BF groups

**Fig. 4:**
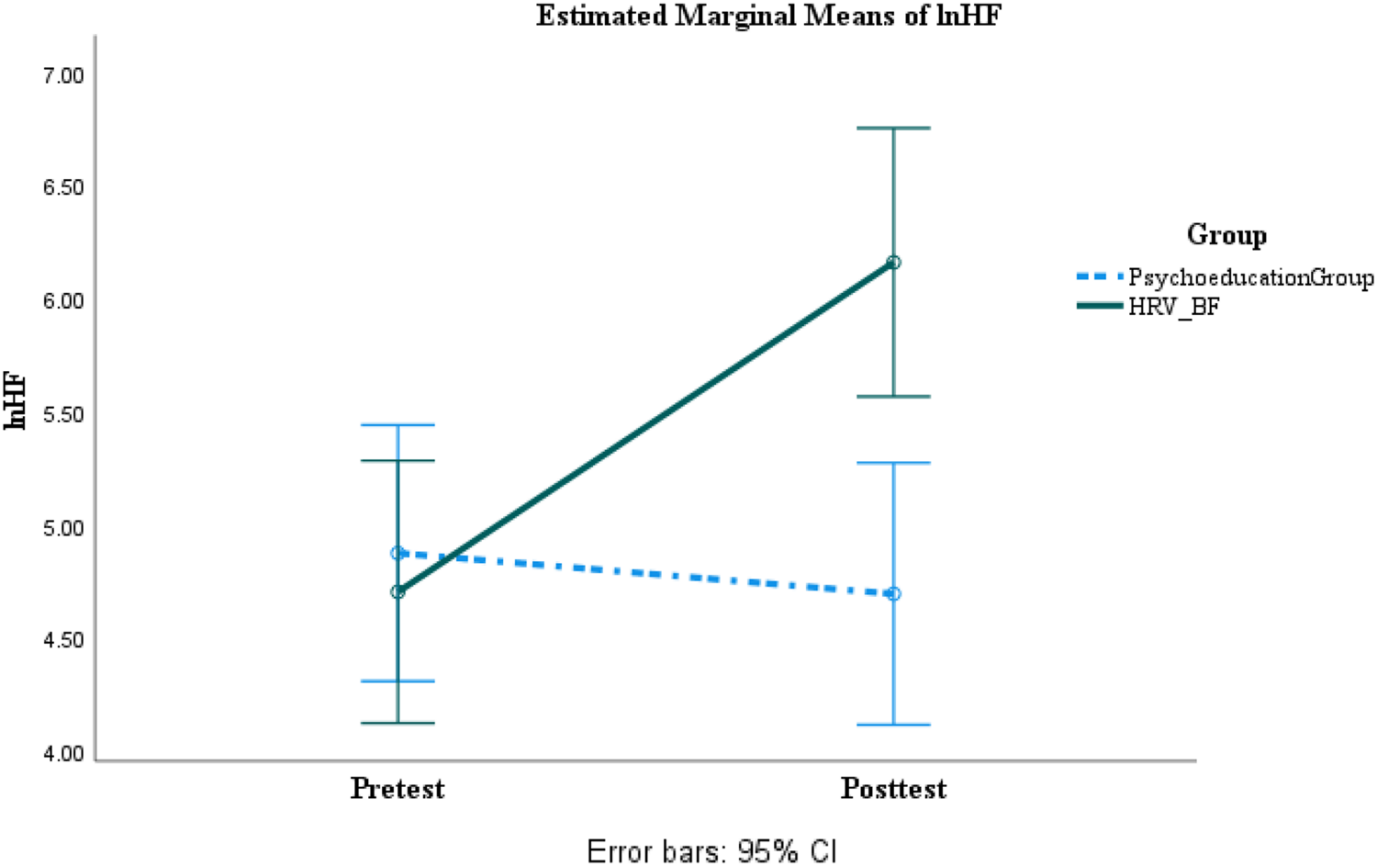
lnHF changes across time between the psychoeducation and the HRV-BF groups

A series of two-way ANOVAs with the group as a between factor and times as within factor were run. As can be seen in Tables 6 and 7. For neuropsychological functioning measures, the HRV-BF group showed significant improvement with large effect sizes in executive functions, medium to large effect sizes in information processing, and small to large effect sizes in verbal memory while the psychoeducation group showed no significant change (Figs. 2, 3 and 4) (Supplemental Content 1–16). In terms of post-concussion symptoms, anxiety, depression, and irritability, the HRV-BF group showed significant improvement with large effect sizes, while the psychoeducation group did not show significant change. For autonomic nervous system functioning, the HRV-BF group showed significant improvement with large effect sizes in HRV while the psychoeducation group showed no significant improvement.

**Table 6:** 2 × 2 Repeated Measures ANOVA for Neuropsychological Measures at Pre- and Post-treatment between The Psychoeducation and HRV-BF Group

Variables	Psychoeducation (n = 21)		HRV-BF (n = 20)		*F*			*η*^*2*^_*p*_ / d	The post hoc comparison
	Pre-treatment	Post-treatment	Pre-treatment	Post-treatment	Group (*p*)	Time (*p*)	Group × Time (*p*)		
Executive Function									
TFAB	14.29 (2.33)	14.05 (2.22)	13.65 (2.18)	15.90 (1.12)	1.08 (.306)	17.29*** (< .001)	26.44*** (< .001)	.40 / 1.58	Group: HRV-BF > psychoeducation at post-test Time: post-test > pre-test in HRV-BF
SVF	37.29 (9.14)	34.14 (9.03)	33.10 (8.17)	40.45 (10.02)	.15 (.701)	7.74** (.008)	48.17*** (< .001)	.55 / 2.15	Group: HRV-BF > psychoeducation at post-test Time: post-test > pre-test in HRV-BF
TMT-B	82.87 (53.74)	81.89 (53.33)	83.33 (36.97)	56.79 (21.57)	.89 (.352)	11.74** (.001)	10.11** (.003)	.21 / .94	Time: post-test < pre-test in HRV-BF
TDEX	22.38 (12.03)	26.67 (12.54)	23.60 (11.39)	16.55 (8.13)	1.80 (.188)	1.60 (.213)	26.95*** (< .001)	.41 / 1.59	Group: HRV-BF < psychoeducation at post-test Time: post-test < pre-test in HRV-BF
Information Processing									
PASAT	72.24 (21.18)	73.90 (18.15)	74.60 (14.94)	88.95 (9.68)	3.00 (.091)	36.69*** (< .001)	23.01*** (< .001)	.37 / 1.47	Group: HRV-BF > psychoeducation at post-test Time: post-test > pre-test in HRV-BF
TMT-A	36.21 (11.30)	31.16 (11.57)	39.51 (15.59)	27.18 (8.75)	.01 (.920)	28.47*** (< .001)	5.00* (.031)	.11 / .62	Time: post-test < pre-test in HRV-BF and psychoeducation
Verbal Memory									
Verbal Learning									
Immediate free recall	52.90 (6.35)	52.33 (7.51)	51.30 (6.84)	55.80 (4.42)	.24 (.628)	10.48** (.002)	17.47*** (< .001)	.31 / 1.27	Time: post-test > pre-test in HRV-BF
Delayed Free recall	2.19 (1.54)	2.71 (1.76)	2.20 (1.58)	4.20 (1.32)	2.77 (.104)	44.46*** (< .001)	15.21*** (< .001)	.28 / 1.18	Group: HRV-BF > psychoeducation at post-test Time: post-test > pre-test in HRV-BF and psychoeducation
Cued recall	3.33 (1.59)	3.90 (1.84)	3.30 (1.81)	5.65 (.67)	4.26* (.046)	32.88*** (< .001)	12.19** (.001)	.24 / 1.04	Group: HRV-BF > psychoeducation at post-test Time: post-test > pre-test in HRV-BF
Recognition	26.67 (3.15)	27.67 (2.74)	26.95 (2.44)	29.25 (1.02)	2.59 (.116)	10.21** (.003)	1.58 (.216)	.04 /.24	Group: HRV-BF > psychoeducation at post-test Time: post-test > pre-test in HRV-BF
Symptom /Emotional Function									
CPCS	12.19 (8.81)	11.67 (8.11)	15.40 (10.52)	2.80 (5.19)	1.35 (.252)	46.00*** (< .001)	38.95*** (< .001)	.50 / 1.92	Group: HRV-BF < psychoeducation at post-test Time: post-test < pre-test in HRV-BF
BAI	6.90 (6.64)	5.86 (4.78)	8.55 (6.85)	1.25 (1.52)	1.02 (.319)	25.05*** (< .001)	14.06*** (< .001)	.27 / 1.13	Group: HRV-BF < psychoeducation at post-test Time: post-test < pre-test in HRV-BF
BDI	9.00 (6.57)	10.67 (7.98)	7.75 (8.30)	2.80 (4.40)	5.06* (.030)	4.07 (.051)	16.51*** (< .001)	.30 / 1.23	Group: HRV-BF < psychoeducation at post-test Time: post-test < pre-test in HRV-BF
NTUIS	49.38 (20.55)	55.48 (19.68)	49.85 (12.79)	39.20 (9.13)	2.72 (.107)	1.66 (.206)	22.38*** (< .001)	.37 / 1.44	Group: HRV-BF < psychoeducation at post-test Time: post-test < pre-test in HRV-BF

*P < .05, **P < .01, *** *P* < .001

**Table 7:** 2 × 2 Repeated Measures ANOVA for HRV Indices at Pre- and Post-treatment between The Psychoeducation and HRV-BF Group

Variables	Psychoeducation (n = 21)		HRV-BF (n = 20)		*F*			*η*^*2*^_*p*_ / d	The post hoc comparison
	Pre-treatment	Post-treatment	Pre-treatment	Post-treatment	Group (*p*)	Time (*p*)	Group × Time (*p*)		
SDNN (ms)	22.74 (11.61)	21.11 (12.16)	21.09 (8.65)	36.79 (11.58)	4.54* (.040)	40.80*** (< .001)	61.89*** (< .001)	.61 / 2.44	Group: HRV-BF > psychoeducation at post-test Time: post-test > pre-test in HRV-BF
RMSSD (ms)	19.79 (11.95)	19.24 (12.39)	19.06 (7.81)	36.80 (12.05)	6.73* (.013)	41.07*** (< .001)	46.42*** (< .001)	.54 / 2.11	Group: HRV-BF > psychoeducation at post-test Time: post-test > pre-test in HRV-BF
lnHF (ms^2^)	4.87 (1.40)	4.69 (1.56)	4.70 (1.15)	6.16 (.98)	2.90 (.096)	20.35*** (< .001)	33.43*** (< .001)	.46 / 1.78	Group: HRV-BF > psychoeducation at post-test Time: post-test > pre-test in HRV-BF
lnLF (ms^2^)	5.28 (1.10)	4.90 (1.51)	5.12 (1.02)	5.82 (.89)	1.32 (.258)	1.17 (.286)	13.84*** (< .001)	.26 /1.12	Group: HRV-BF > psychoeducation at post-test Time: post-test > pre-test in HRV-BF
LF/HF (%)	2.13 (1.80)	2.01 (1.88)	2.53 (2.74)	.90 (.66)	.48 (.491)	8.23** (.007)	6.03* (.019)	.13 / .70	Group: HRV-BF < psychoeducation at post-test Time: post-test < pre-test in HRV-BF

^*^P < .05, **P < .01, *** *P* < .001

Because randomization failed for the one variable, LOC, we reran the analyses as a three-way ANOVA with LOC as a second between variable (Group by LOC by Time). No three-way interactions were found for any of the measures and the Group by time interactions remained significant. Thus, it appears that the randomization failure did not affect the results. Further analyses using PROCESS revealed that the measures related to attention did not mediate the effect between HRV-BF and neuropsychological functioning. These results are included in the supplemental section (Supplemental Content 37–38).

## Discussion

This study was the first randomized control study in support of self-regulation training using a psychophysiological intervention based on HRV-BF for those afflicted with mild traumatic brain injury. The use of HRV-BF was used to improve psychophysiological and neuropsychological functioning in individuals with mild traumatic brain injury. Several studies have demonstrated that HRV is reduced in individuals who have suffered a traumatic brain injury and that improvements in HRV are concurrent with functional recovery. In our results, compared to the psychoeducation group, the HRV-BF group had significant improvements in executive functioning, information processing, verbal memory, and emotional functions (anxiety, depression, and irritability).

The current study results are consistent with previous findings in non-TBI populations, showing that a positive effect of HRV-BF may be observed on executive function, including attention, inhibition, and working memory (Bruin et al., 2016; Ginsberg et al., 2010; Jester et al., 2019; Sutarto et al., 2013). However, the results of past studies have not demonstrated a direct effect of HRV-BF training on improved neuropsychological performance in TBI populations.

In terms of emotional disturbances, our findings are consistent with previous findings and showed that participants in the HRV-BF group had improvement in emotional regulation in patients with TBI, including reducing depression (Lagos et al., 2013; Wearne et al., 2021). In contrast to previous studies, the present study also found that HRV-BF can also reduce anxiety and irritability in addition to reducing symptoms of depression for patients with TBI.

The occurrence of PCS is common following a mild traumatic brain injury. Our results indicated that HRV-BF participants displayed significantly lower levels of PCS compared to those in the psychoeducation group between pre- and post-tests, which is consistent with the previous preliminary case study that indicated clinically significant improvements in post-concussive symptoms in a concussed patient (Lagos et al., 2013).

As for HRV indices, the results of this study were consistent with Lagos et al. and Kim et al. who found increased LF and LF/HF of HRV from pre-and posttest in the HRV-BF group (Kim et al., 2015; Lagos et al., 2013). Our study also showed that SDNN, RMSSD, and HF increased significantly in the HRV-BF group, which indicated that the training carry-over effects directly on the intervention of HRV-BF on vagal-cardiac activity (Lehrer & Gevirtz, 2014; Berntson et al., 2005).

This study had a relatively small sample size and targeted patients with mild traumatic brain injury (mTBI), so we cannot draw definitive conclusions and/or make suggestions for all forms of brain injury. It is also possible that the dose–response of the study interventions may differ between the psychoeducational group and the HRV-BF group. Each participant met with the researcher during the pre-test and post-test after brain injury. The HRV-BF group also met with the researcher for training sessions during the 10-session period. Participants in the HRV-BF group may be more motivated and more compliant than those in the psychoeducation group, which may affect the effectiveness of the intervention. However, for the purpose of reducing the influence of rapport between researchers and participants on the standardization of pretest and posttest administration, the researchers who administered the pretest or posttest were different from those who conducted the interventions. Future studies would benefit by using a sham biofeedback procedure such as that utilized by Bachman et al., (2022). Additionally, more people in the HRV-BF group experienced loss of consciousness (LOC) following head injuries, indicating a more severe brain injury. However, participants in the HRV-BF group achieved greater functional improvements after the intervention, demonstrating that HRV-BF may be an effective rehabilitation method for mild traumatic brain injury that enhances neuropsychological and physiological function.

Despite the limitations of this small sample size study, this study contributes significantly to the mental health field with regard to understanding the benefits of cutting-edge treatment for mild brain injury patients in order to reduce the negative impact of these injuries on public health and patient wellbeing. In regards to the satisfaction of the participants with their treatment, the researchers did not formally ask the participants to provide written feedback, however, many participants in the HRV-BF group provided positive verbal feedback. For example, some participants told researchers and neurosurgeons that after HRV-BF intervention, their post-concussion symptoms significantly improved and helped them to improve their productivity after returning to work.

In this study, HRV-BF interventions were found to modulate central-peripheral nervous system function in mTBI patients by enhancing HRV, stimulating the vagal nerve, and balancing sympathetic and parasympathetic activity. Additionally, HRV-BF interventions contributed to improvement in cognitively challenging tasks within a short timeframe. A particular significance of this study is its demonstration of the effectiveness of HRV-BF training in developing physiological and neuropsychological outcomes quickly without requiring significant cognitive effort. The HRV-BF intervention may be clinically feasible for rehabilitation in patients with mTBI. Further study with a larger sample size and long-term follow-up can be conducted to verify whether those with better recovery have better long-term brain injury prognosis, including fewer PCS, better cognitive functions, or higher HRV.

### Supplementary Information

Below is the link to the electronic supplementary material.

Supplementary file1 (DOCX 2698 KB)
