# Supplementary material for: Heart Rate Variability Biofeedback for Mild Traumatic Brain Injury: A Randomized-Controlled Study
Source: Appl Psychophysiol Biofeedback. 2023 Jun 19;48(4):405–21. doi: 10.1007/s10484-023-09592-4 (PMC10582136; doi:10.1007/s10484-023-09592-4)
Supplement: Supplementary file 1 — Supplementary file1 (DOCX 2698 KB) [file 10484_2023_9592_MOESM1_ESM.docx]

**Supplemental Content**

**Article Title:** Heart Rate Variability Biofeedback for Mild Traumatic Brain Injury: A Randomized-Controlled Study

**Journal Name:** Applied Psychophysiology and Biofeedback

**Author Name:** Hsueh Chen Lu^a^, M.S.; Richard Gevirtz^a^, Ph.D.; Chi Cheng Yang^d^, Ph.D.; Alexander O. Hauson^a,b,c^, Ph.D.

**Author affiliations:**

^a^California School of Professional Psychology at Alliant International University, Clinical Psychology PhD Program, San Diego, CA, USA

^b^Department of Psychiatry, University of California San Diego, La Jolla, CA, USA

^c^Institute of Brain Research and Integrated Neuropsychological Services (iBRAINS.org), San Diego, CA, USA

^d^Department of Psychology, National Chengchi University, Taipei, Taiwan

**The corresponding author:** Richard Gevirtz, Ph.D. ([Rgevirtz@alliant.edu](mailto:Rgevirtz@alliant.edu))


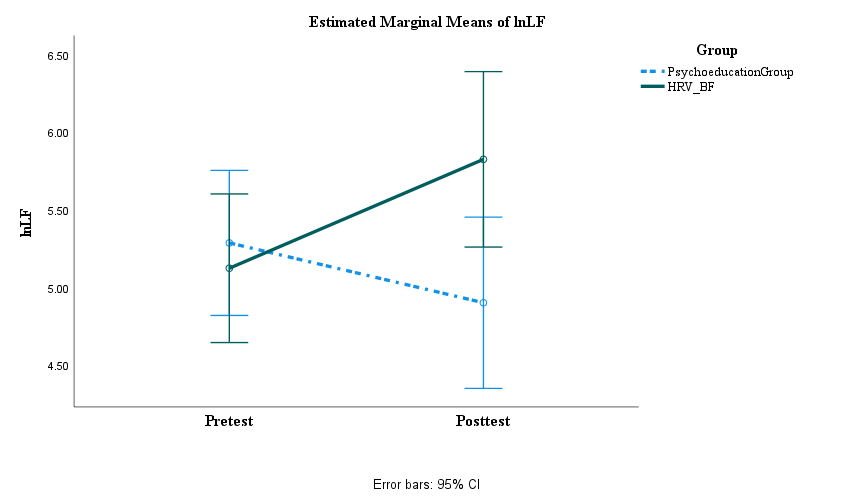


Supplemental Content 1. lnLF changes across time between the psychoeducation and the HRV-BF groups


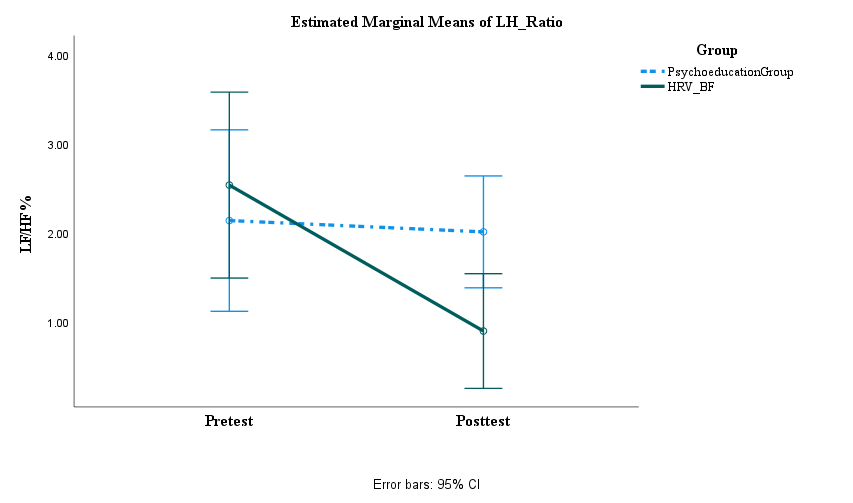


Supplemental Content 2. LF/HF% changes across time between the psychoeducation and the HRV-BF groups


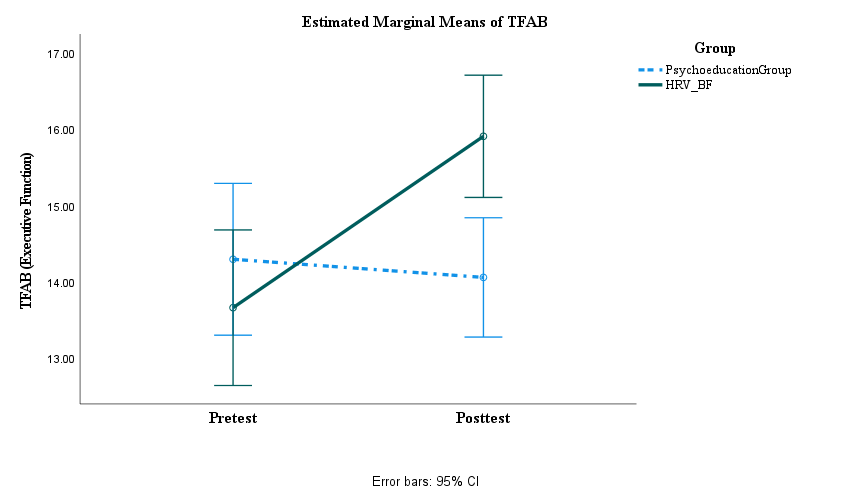


Supplemental Content 3. TFAB changes across time between the psychoeducation and the HRV-BF groups

TFAB = Taiwanese Frontal Assessment Battery


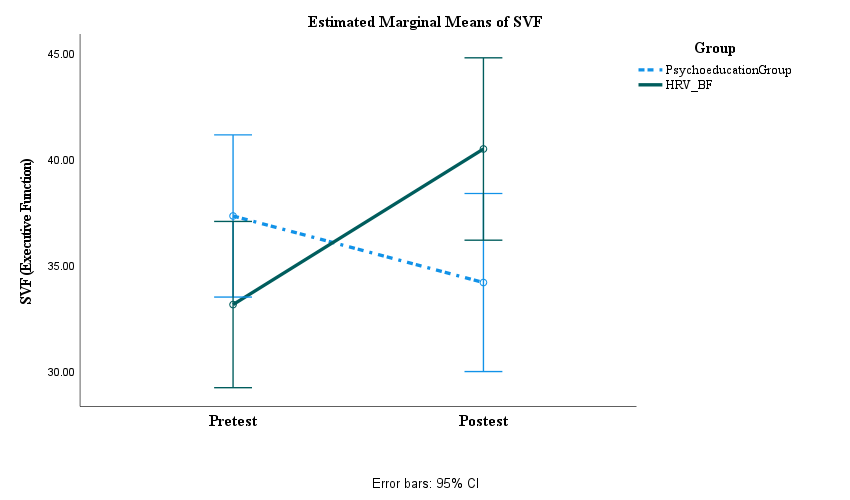


Supplemental Content 4. SVF changes across time between the psychoeducation and the HRV-BF groups

SVF = Semantic Association of Verbal Fluency Test


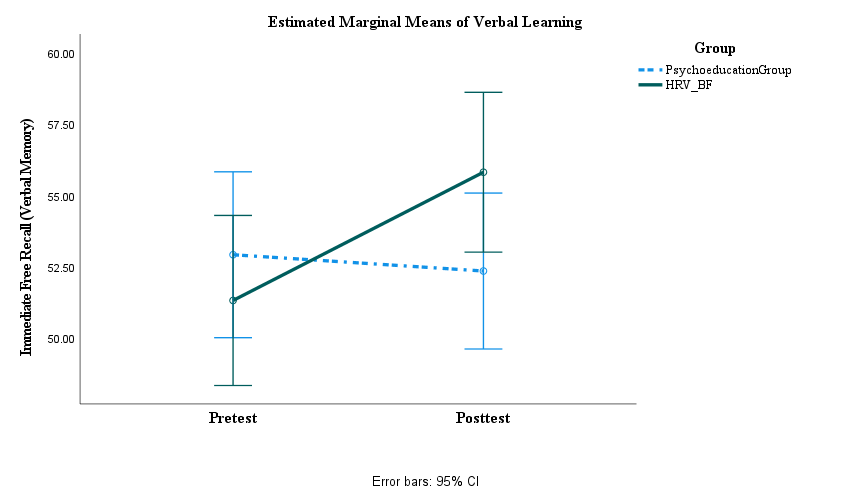


Supplemental Content 5. Immediate free recall changes across time between the psychoeducation and the HRV-BF groups


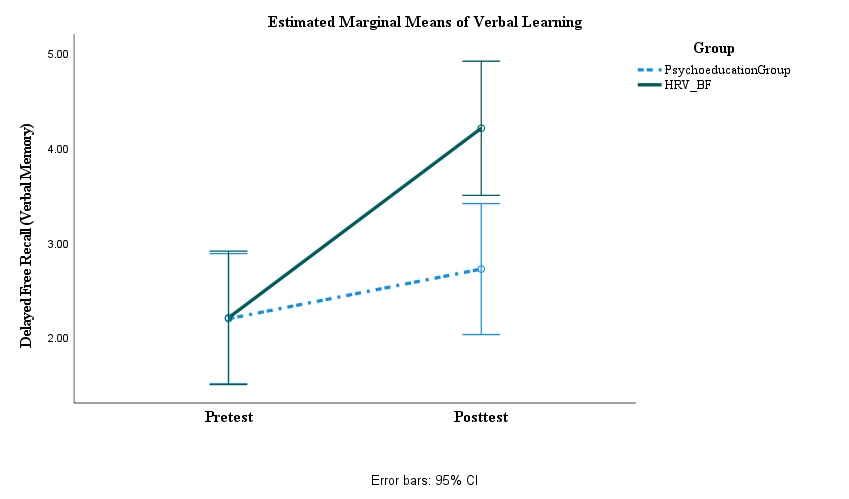


Supplemental Content 6. Delayed free recall changes across time between the psychoeducation and the HRV-BF groups


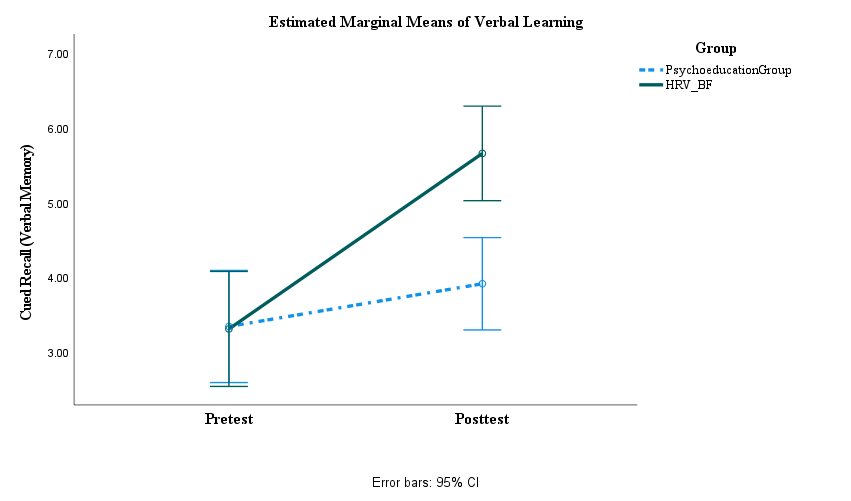


Supplemental Content 7. Cued recall changes across time between the psychoeducation and the HRV-BF groups

***
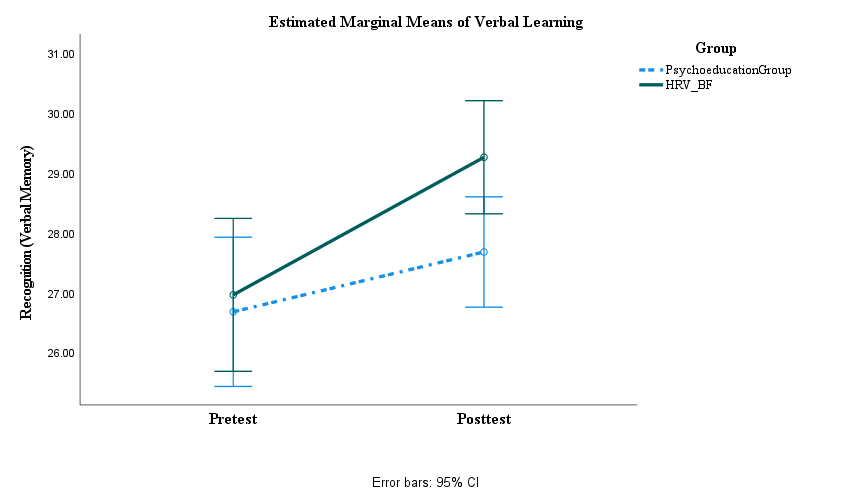
***

Supplemental Content 8. Recognition changes across time between the psychoeducation and the HRV-BF groups


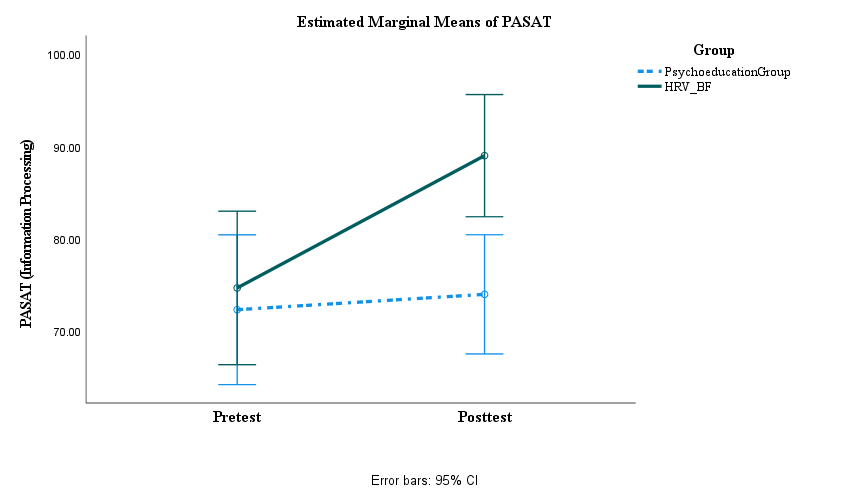


Supplemental Content 9. PASAT changes across time between the psychoeducation and the HRV-BF groups

PSAST = Paced Auditory Serial Addition Test


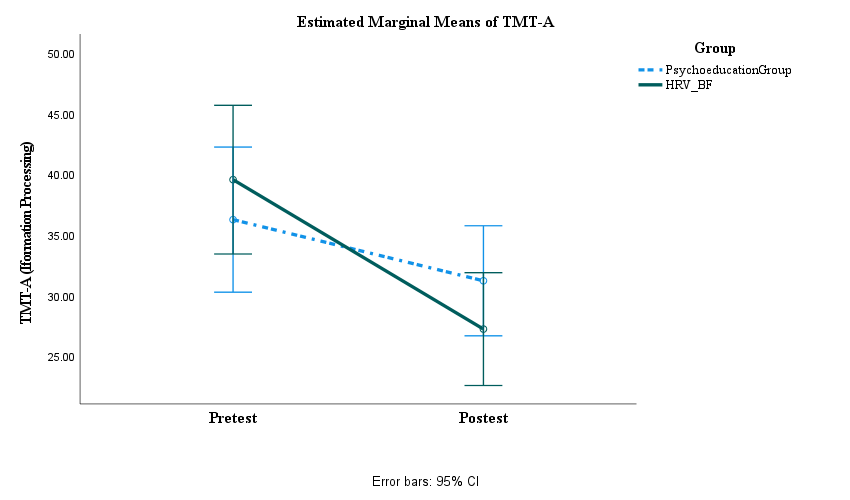


Supplemental Content 10. TMT-A changes across time between the psychoeducation and the HRV-BF groups

TMT-A = Trail Making Test- A


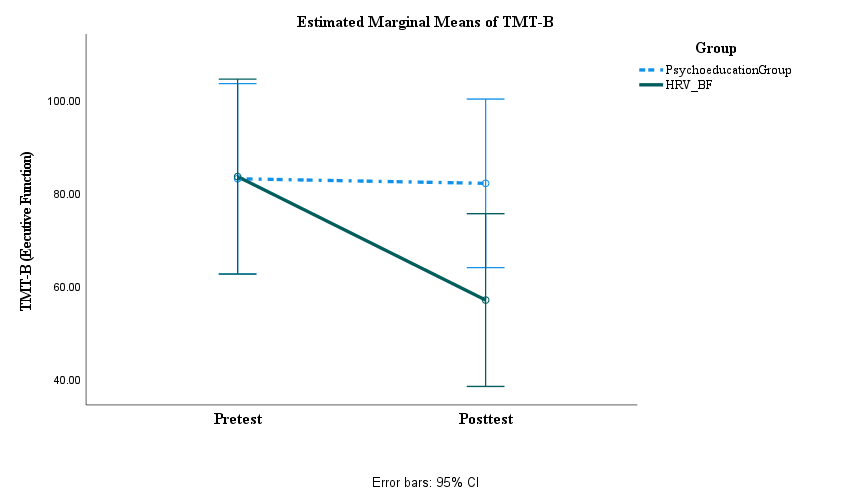


Supplemental Content 11. TMT-B changes across time between the psychoeducation and the HRV-BF groups

TMT-B = Trail Making Test- B


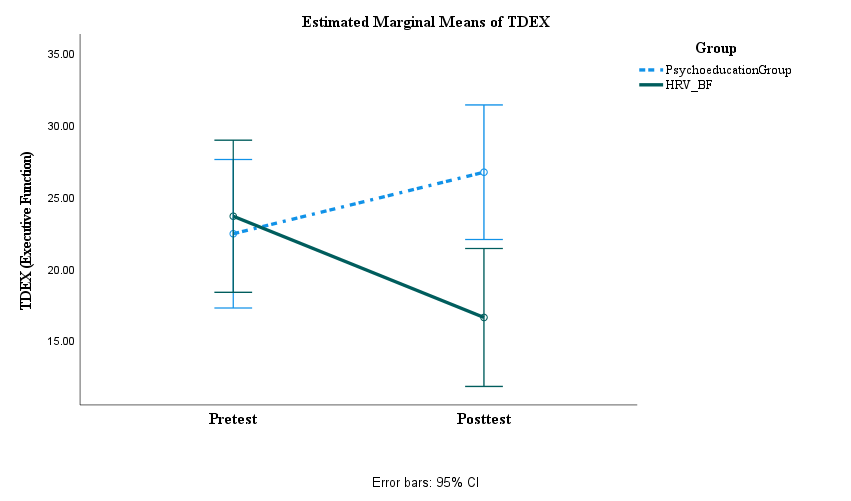


Supplemental Content 12. TDEX changes across time between the psychoeducation and the HRV-BF groups

TDEX = Taiwanese version of Dysexecutive Questionnaire


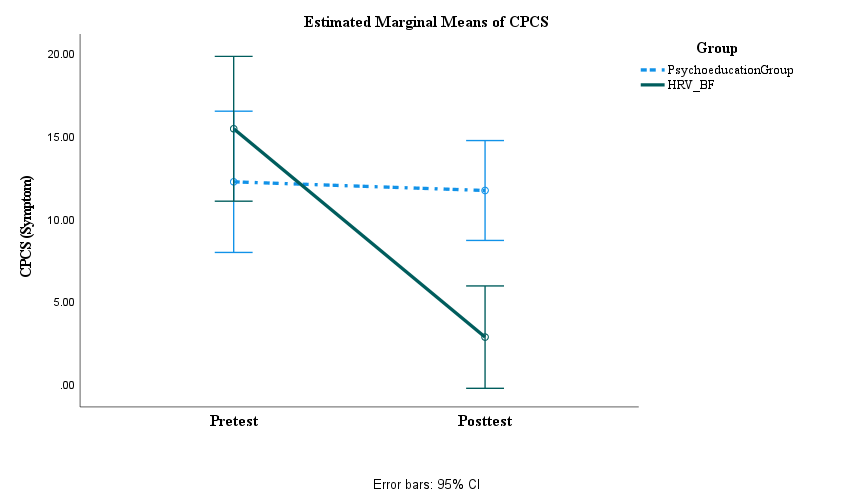


Supplemental Content 13. CPCS changes across time between the psychoeducation and the HRV-BF groups

CPCS = Checklist of Post-concussion Symptoms


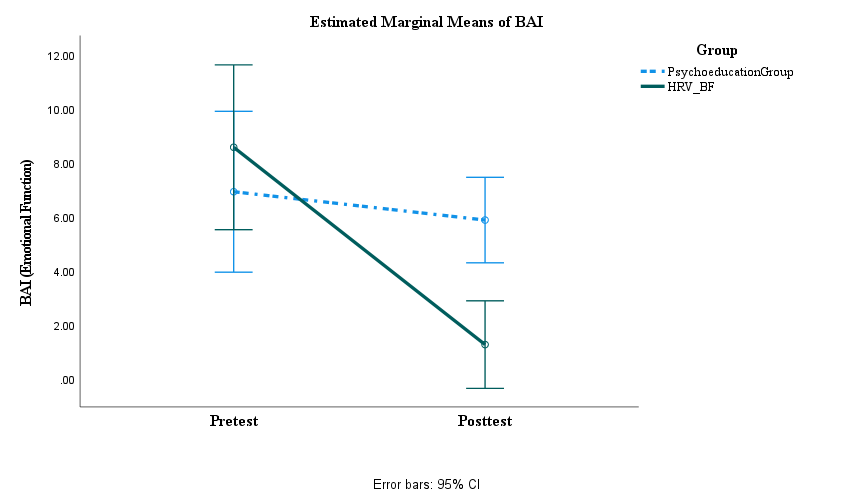


Supplemental Content 14. BAI changes across time between the psychoeducation and the HRV-BF groups

BAI = Beck Anxiety Inventory


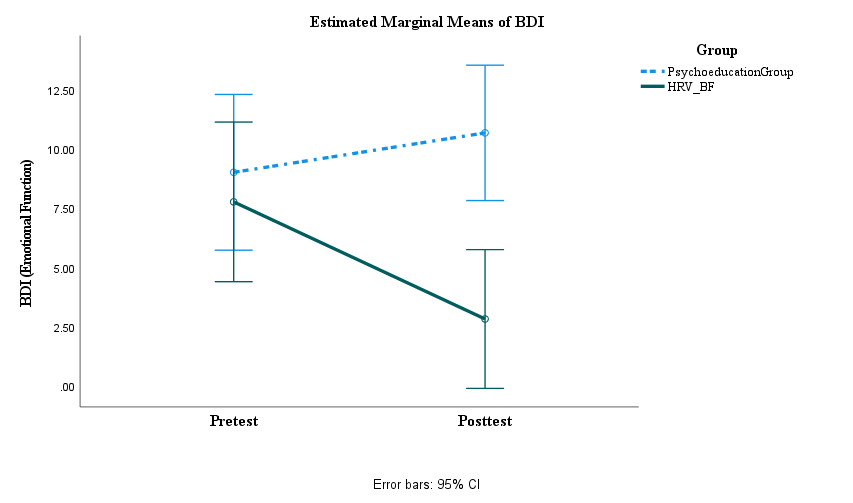


Supplemental Content 15. BDI changes across time between the psychoeducation and the HRV-BF groups

BDI = Beck Depression Inventory


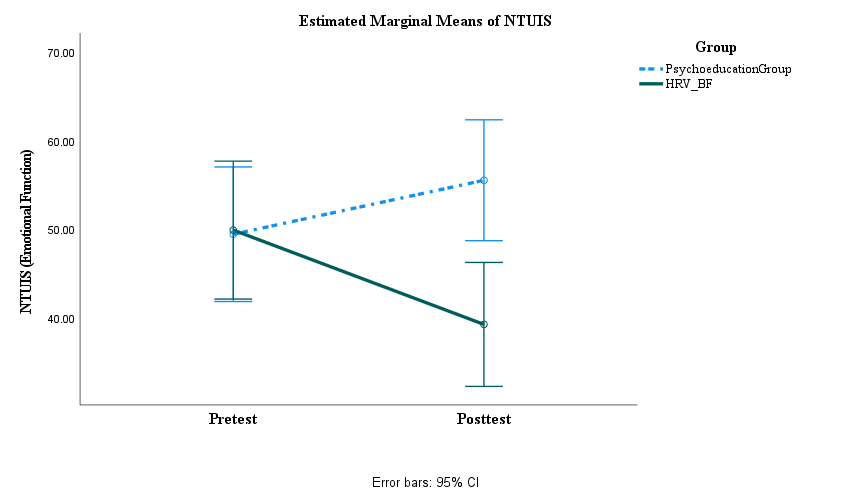


Supplemental Content 16. NTUIS changes across time between the psychoeducation and the HRV-BF groups

NTUIS = The National Taiwan University Irritability Scale

**Spider Plots Showing Each Individual across Time for the Main Outcomes**


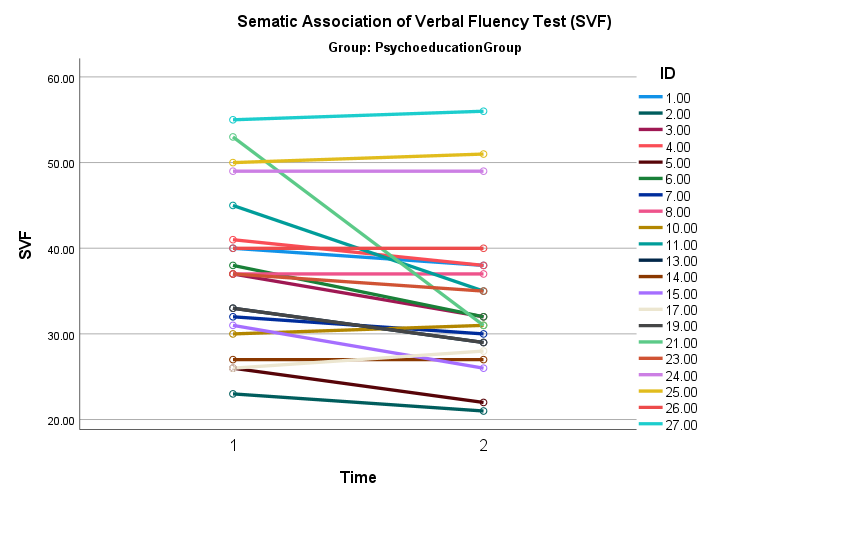


Supplemental Content 17. SVF changes across time in the psychoeducation group

SVF = Semantic Association of Verbal Fluency Test


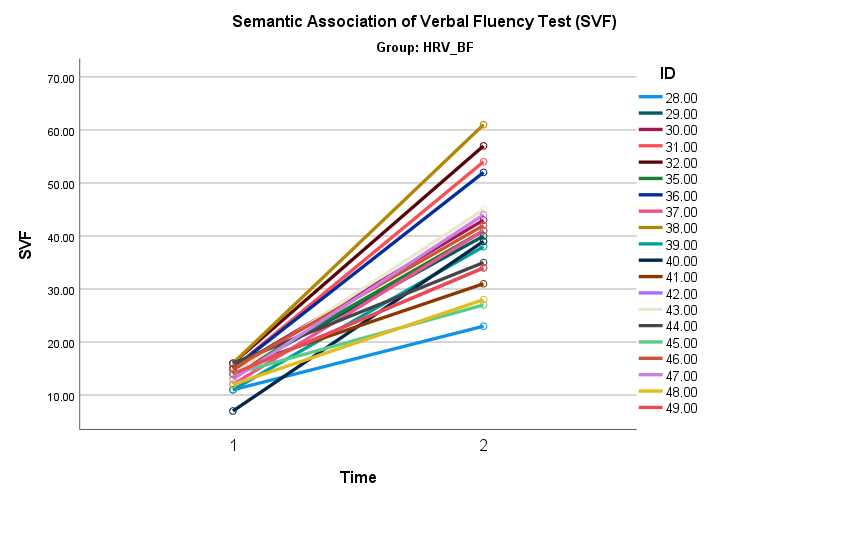


Supplemental Content 18. SVF changes across time in the HRV-BF group

SVF = Semantic Association of Verbal Fluency Test

**
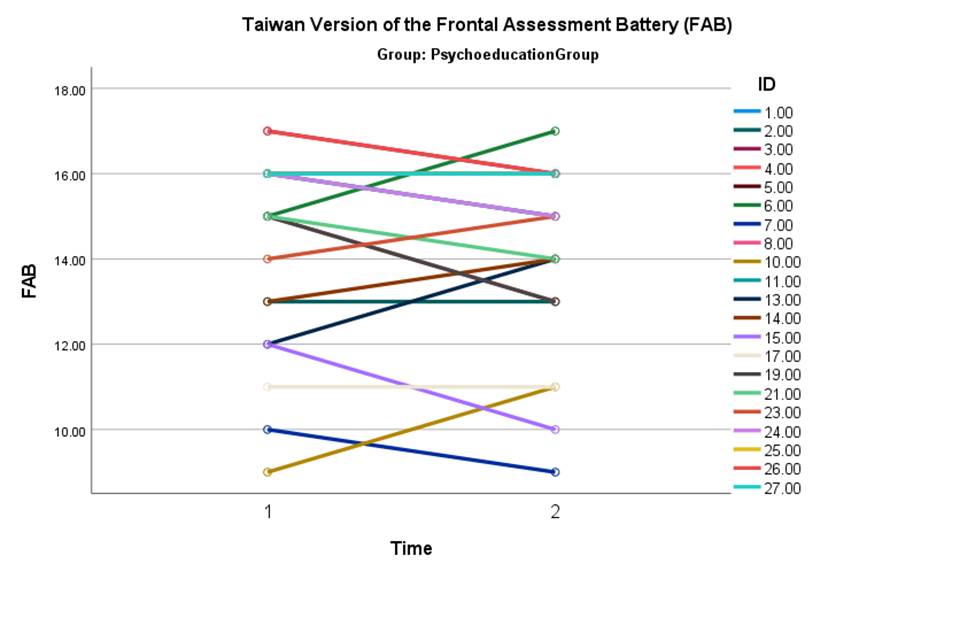
**

Supplemental Content 19. TFAB changes across time in the psychoeducation group

TFAB = Taiwanese Frontal Assessment Battery

**
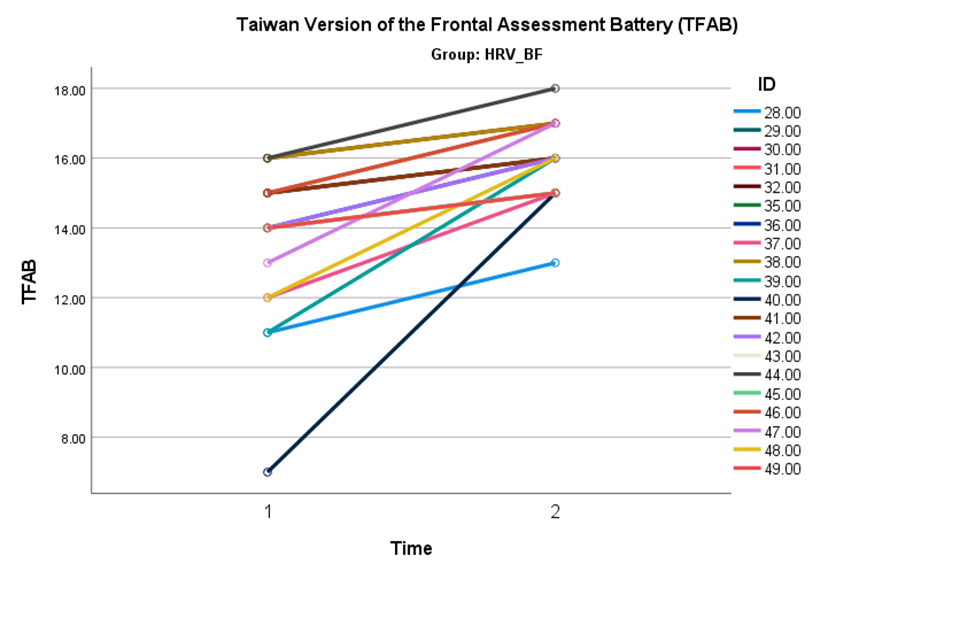
**

Supplemental Content 20. TFAB changes across time in the HRV-BF group

TFAB = Taiwanese Frontal Assessment Battery

**
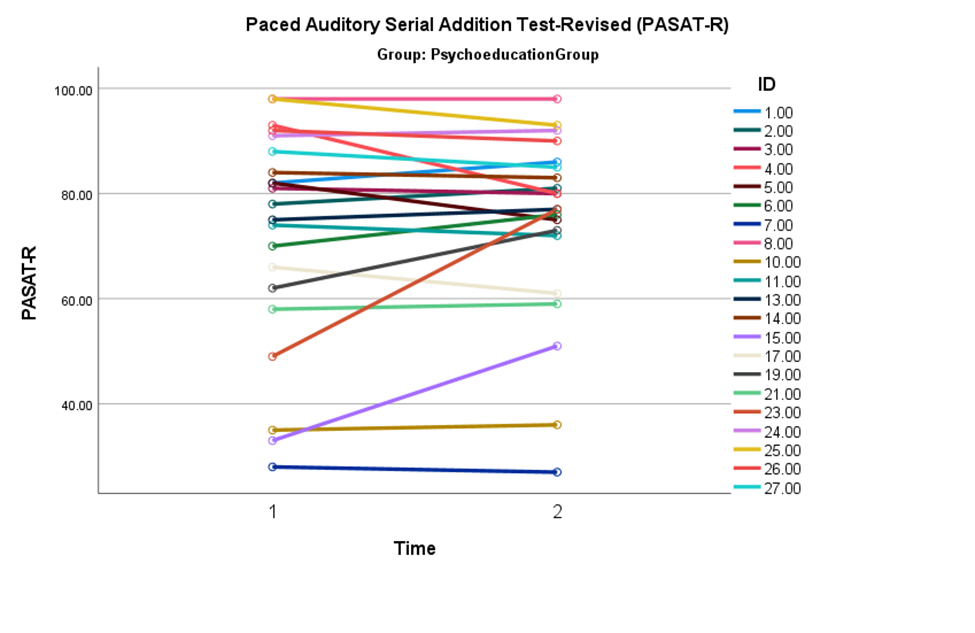
**

Supplemental Content 21. PASAT changes across time in the psychoeducation group

PSAST = Paced Auditory Serial Addition Test

**
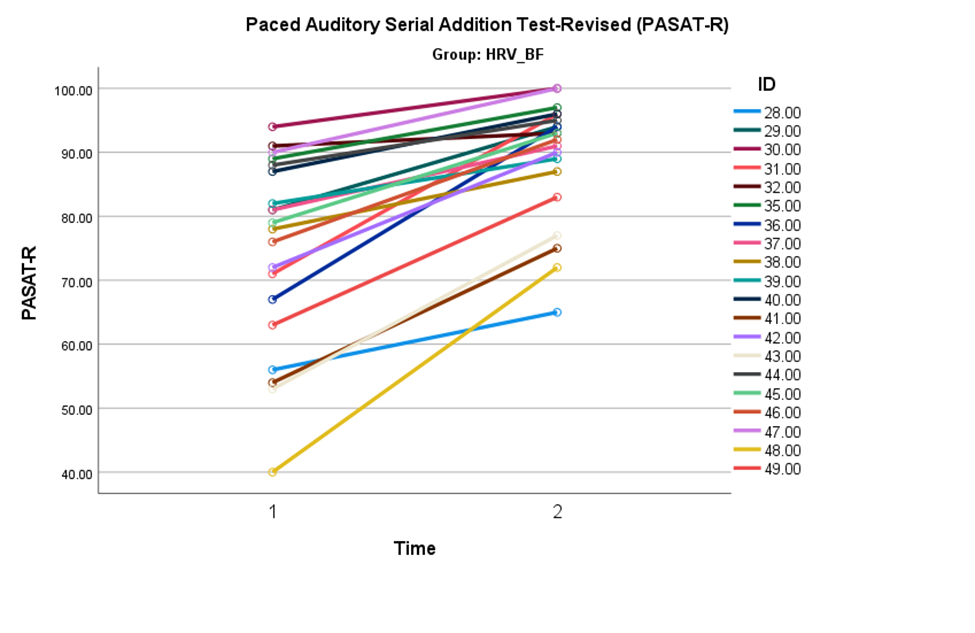
**

Supplemental Content 22. PASAT changes across time in the HRV-BF group

PSAST = Paced Auditory Serial Addition Test

**
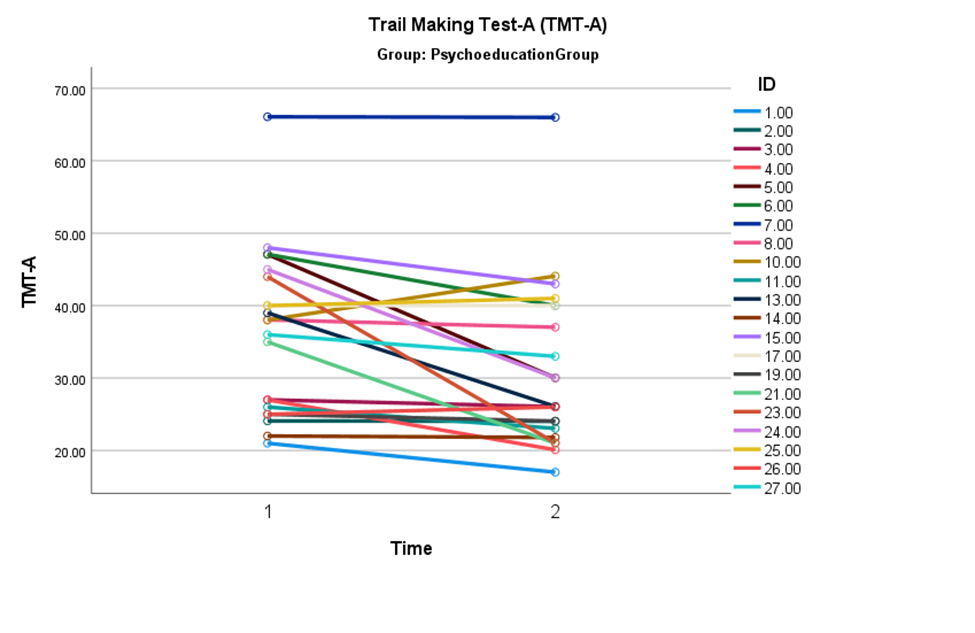
**

Supplemental Content 23. TMT- A changes across time in the psychoeducation group

TMT-A = Trail Making Test- A

**
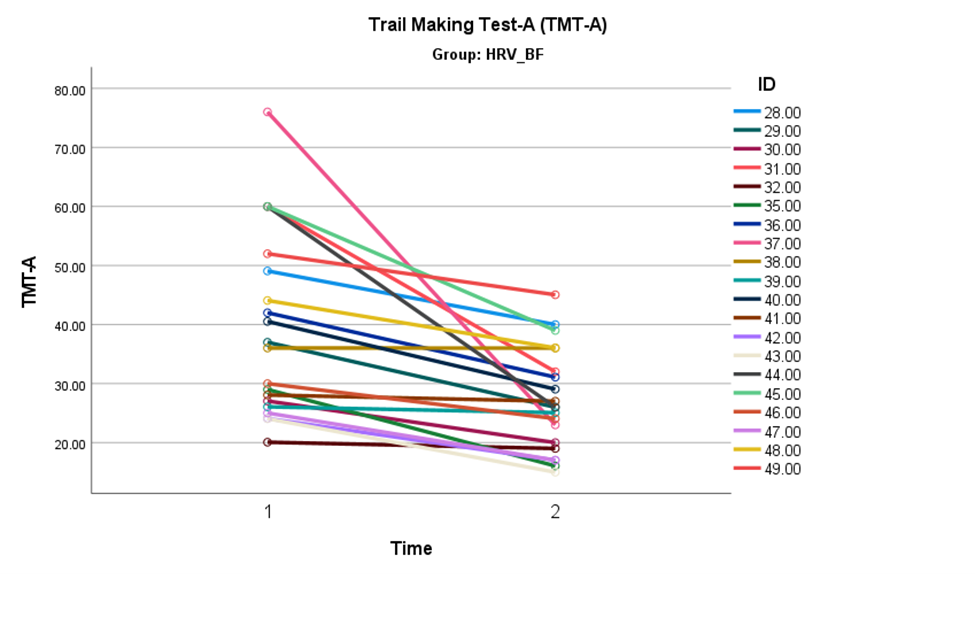
**

Supplemental Content 24. TMT- A changes across time in the HRV-BF group

TMT-A = Trail Making Test- A

**
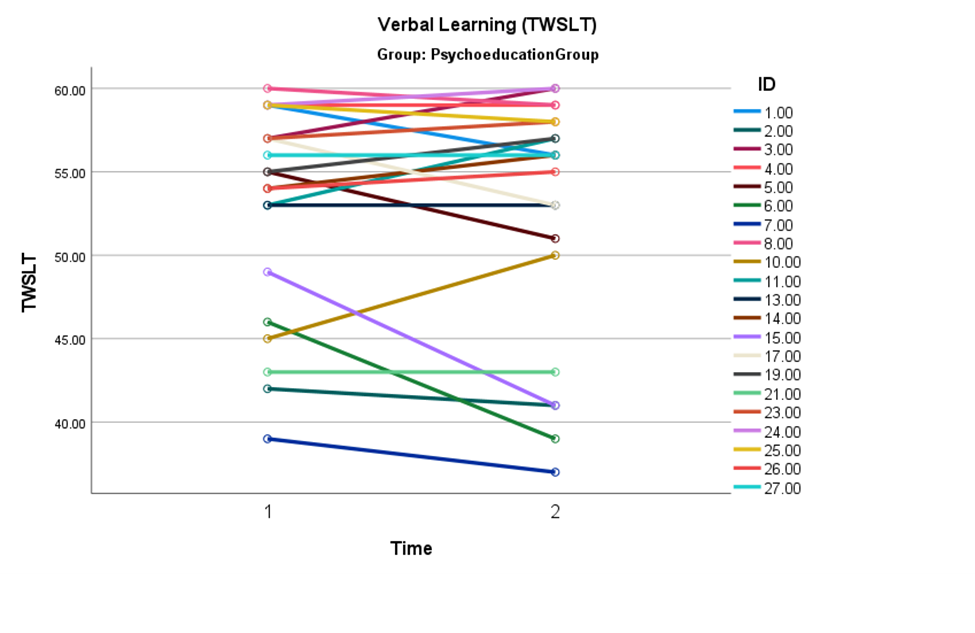
**

Supplemental Content 25. TWSLT- Immediate free recall changes across time in the psychoeducation group

TWSLT = The Taiwanese Version of the Word Sequence Learning Test

**
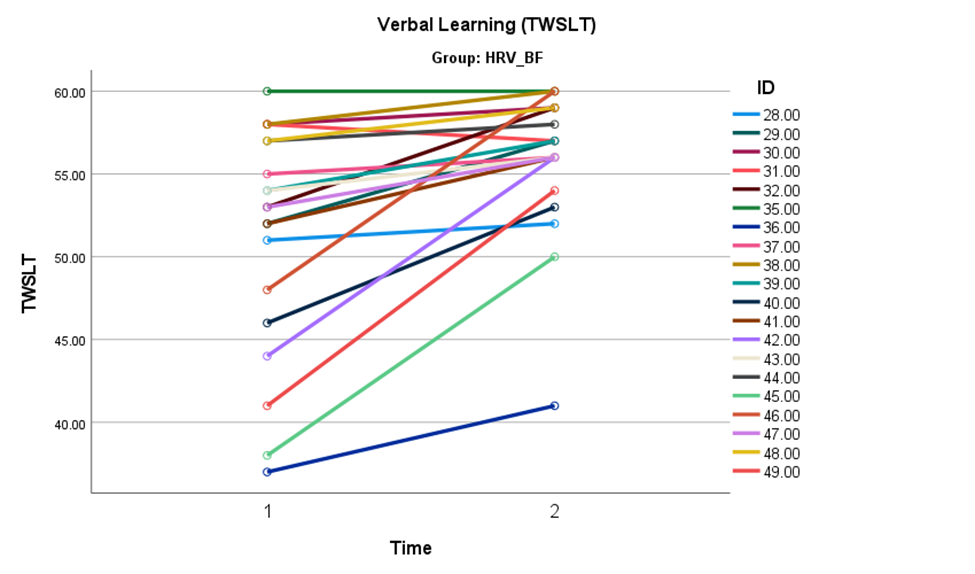
**

Supplemental Content 26. TWSLT- Immediate free recall changes across time in the HRV-BF group

TWSLT = The Taiwanese Version of the Word Sequence Learning Test

**
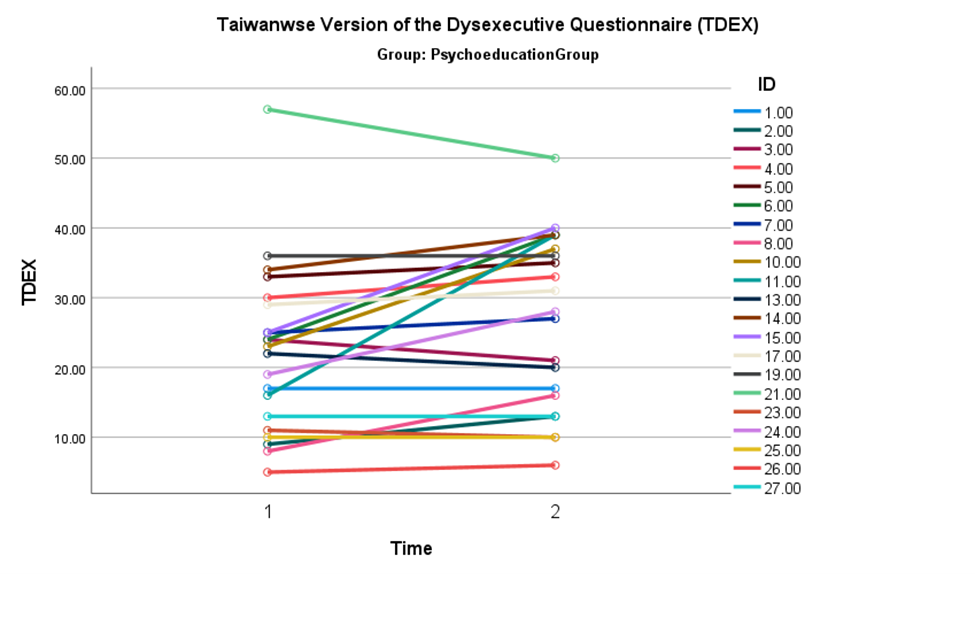
**

Supplemental Content 27. TDEX changes across time in the psychoeducation group

TDEX = Taiwanese version of Dysexecutive Questionnaire

**
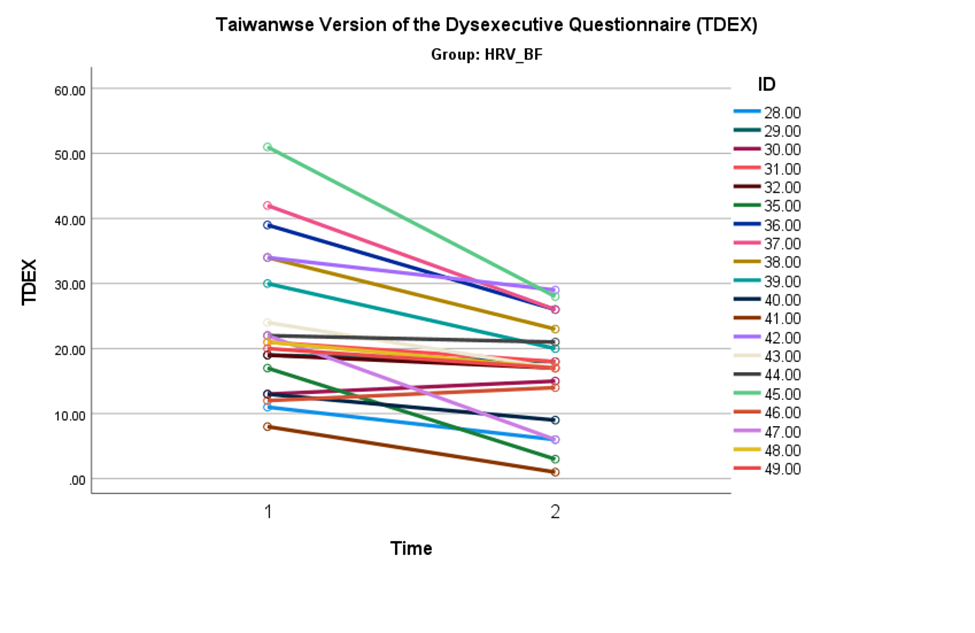
**

Supplemental Content 28. TDEX changes across time in the HRV-BF group

TDEX = Taiwanese version of Dysexecutive Questionnaire

**
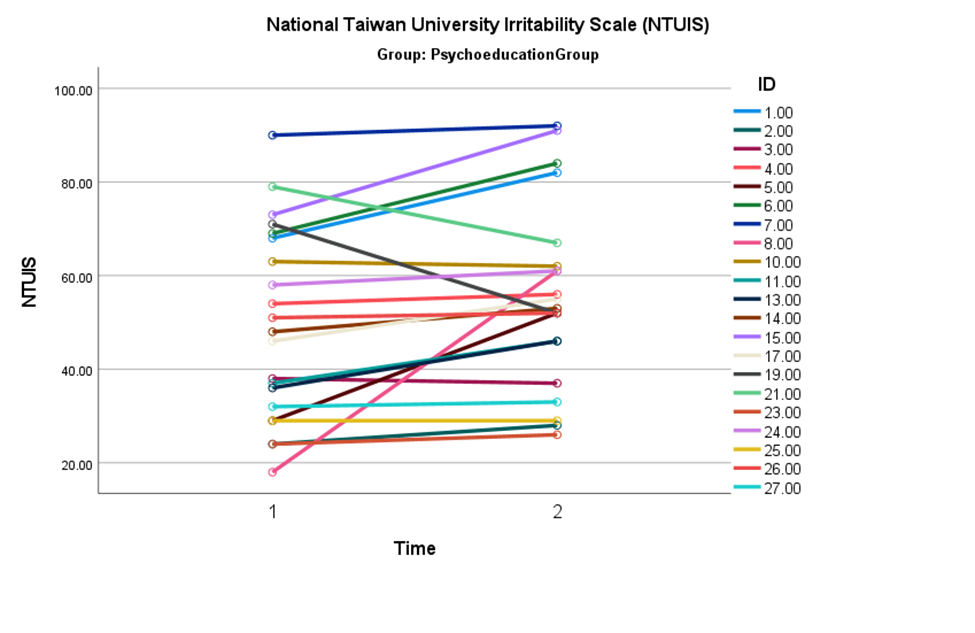
**

Supplemental Content 29. NTUIS changes across time in the psychoeducation group

NTUIS = The National Taiwan University Irritability Scale

**
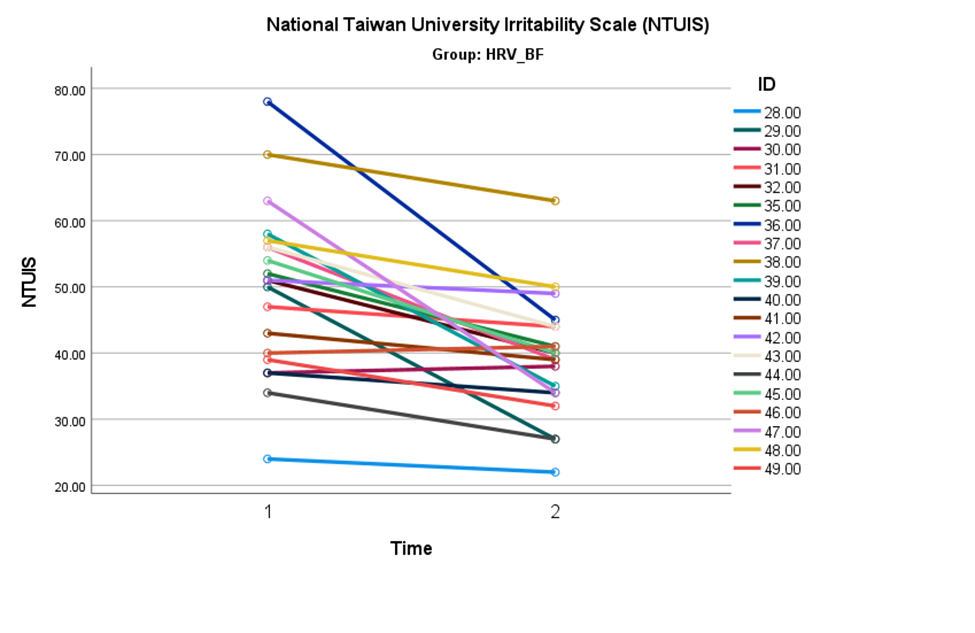
**

Supplemental Content 30. NTUIS changes across time in the HRV-BF group

NTUIS = The National Taiwan University Irritability Scale


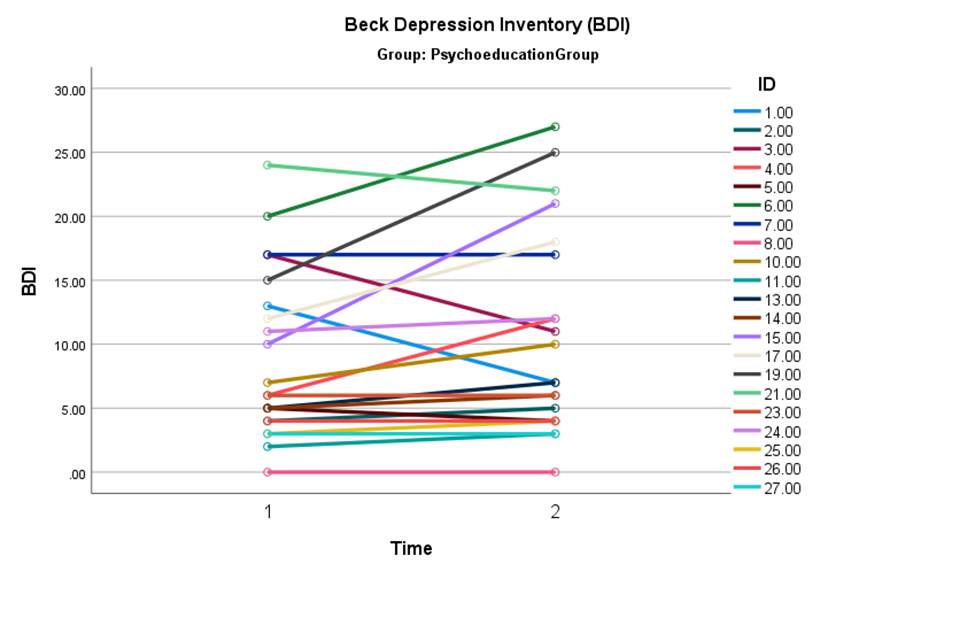


Supplemental Content 31. BDI changes across time in the psychoeducation group

BDI = Beck Depression Inventory


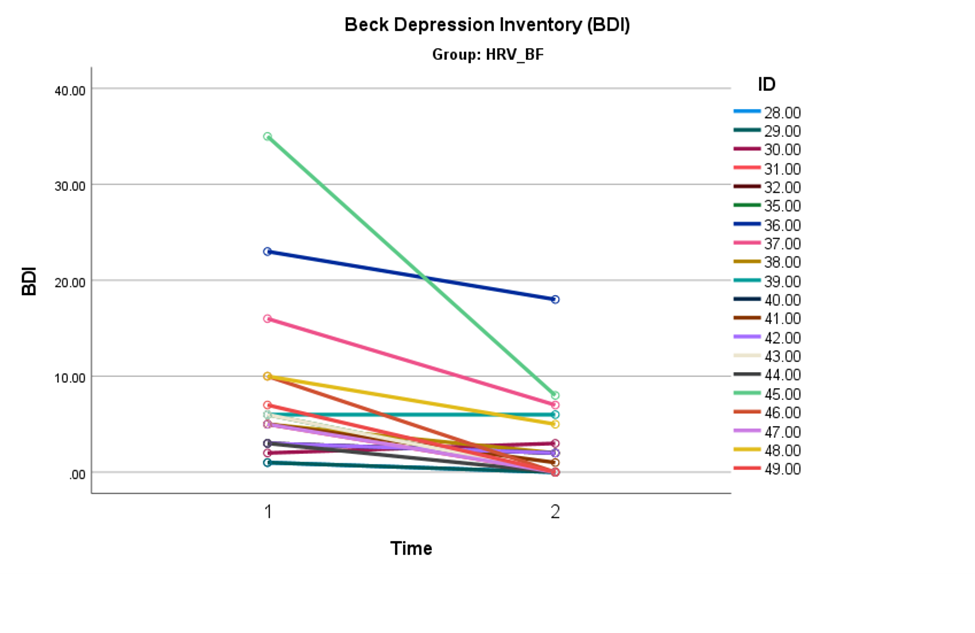


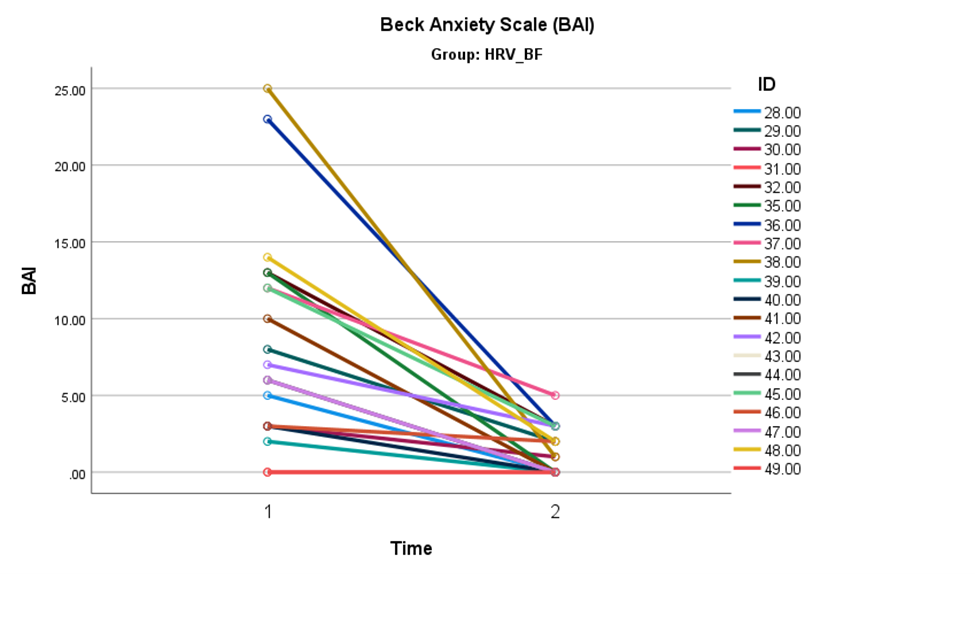


Supplemental Content 32. BDI changes across time in the HRV-BF group

BDI = Beck Depression Inventory


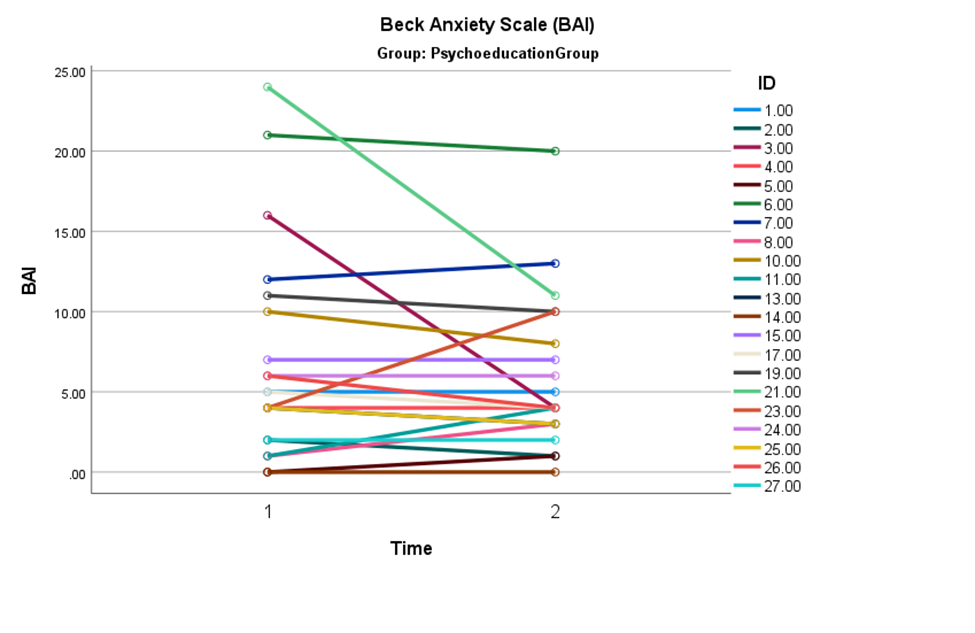


Supplemental Content 33. BAI changes across time in the psychoeducation group

BAI = Beck Anxiety Inventory


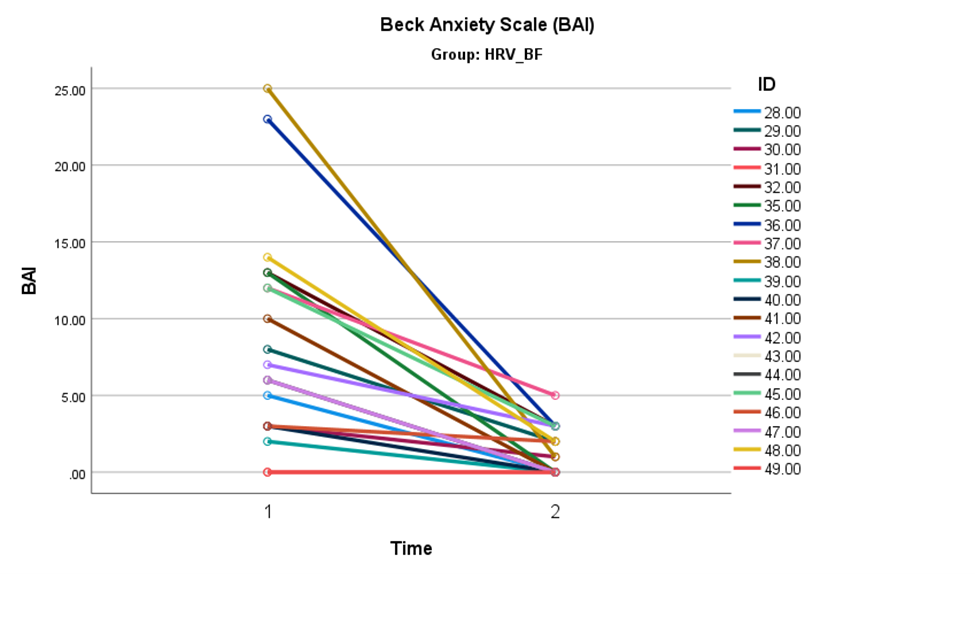


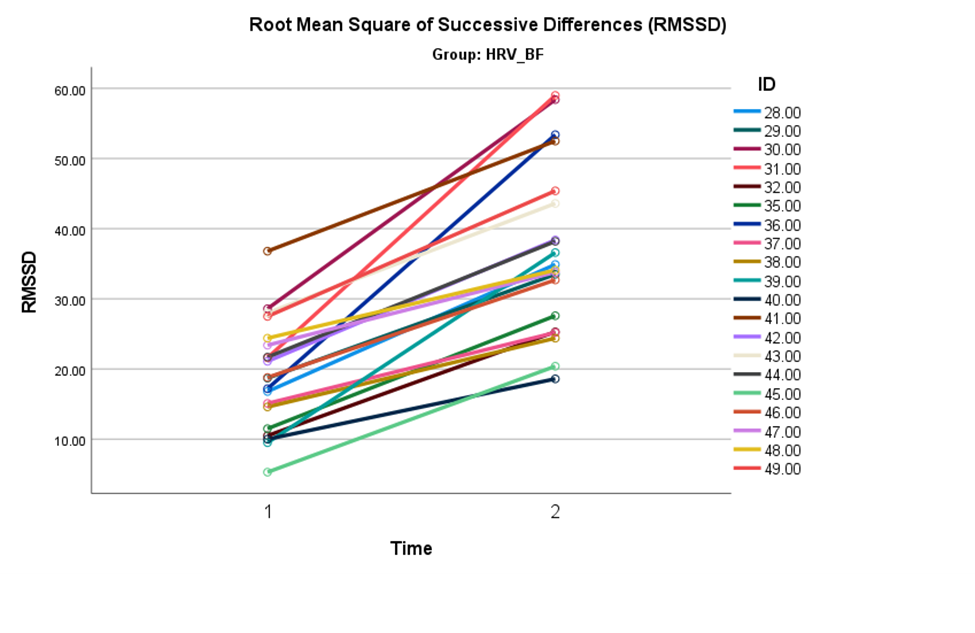


Supplemental Content 34. BAI changes across time in the HRV-BF group

BAI = Beck Anxiety Inventory


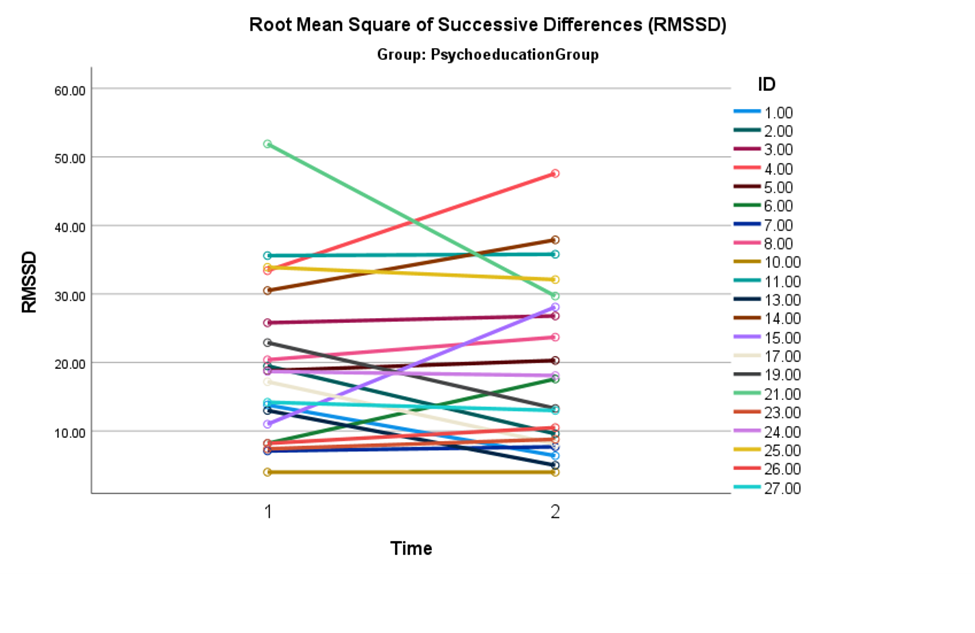


Supplemental Content 35. RMSSD changes across time in the psychoeducation group


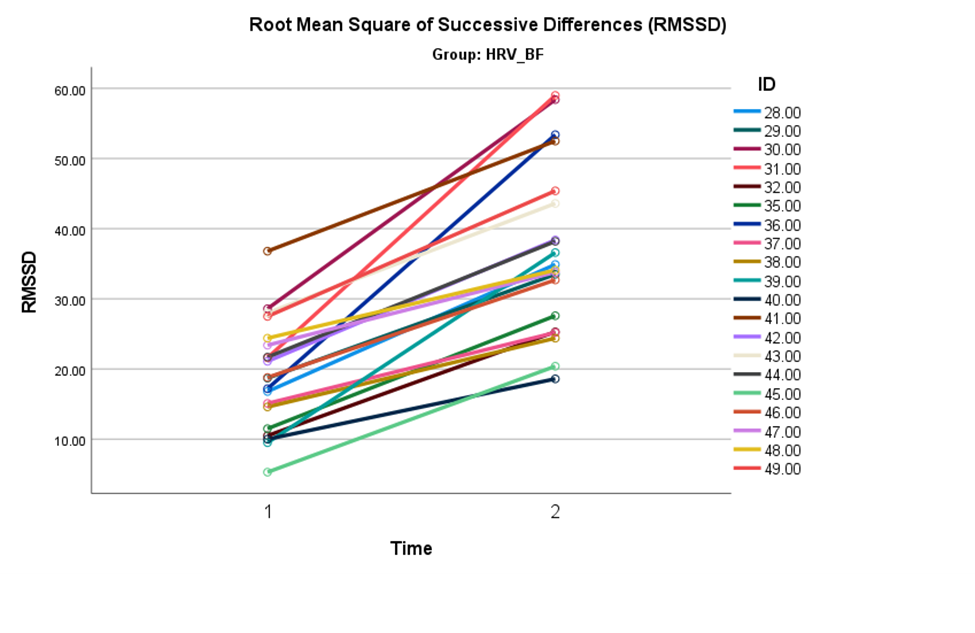


Supplemental Content 36. RMSSD changes across time in the HRV-BF group

Supplemental Content 37. Mediational analysis testing whether Trail Making Test- A mediated the changes in Frontal Assessment Battery.

************** TOTAL, DIRECT, AND INDIRECT EFFECTS OF X ON Y **************

Total effect of X on Y

Effect se t p LLCI ULCI c_ps

2.4881 .4839 5.1415 .0000 1.5092 3.4669 1.2560

Direct effect of X on Y

Effect se t p LLCI ULCI c'_ps

2.4196 .5197 4.6557 .0000 1.3675 3.4718 1.2214

Indirect effect(s) of X on Y:

Effect BootSE BootLLCI BootULCI

Delt_TMT .0685 .1318 -.2554 .2999 CI includes 0, so no evidence of mediation

Partially standardized indirect effect(s) of X on Y:

Effect BootSE BootLLCI BootULCI

Delt_TMT .0346 .0731 -.1251 .1802

*********************** ANALYSIS NOTES AND ERRORS ************************

Supplemental Content 38. Mediational analysis testing whether Trail Making Test- A mediated the changes in Frontal Assessment Battery.

************** TOTAL, DIRECT, AND INDIRECT EFFECTS OF X ON Y **************

Total effect of X on Y

Effect se t p LLCI ULCI c_ps

12.6833 2.6442 4.7967 .0000 7.3349 18.0317 1.2037

Direct effect of X on Y

Effect se t p LLCI ULCI c'_ps

12.0064 2.8267 4.2475 .0001 6.2840 17.7288 1.1395

Indirect effect(s) of X on Y:

Effect BootSE BootLLCI BootULCI

Delt_TMT .6769 1.1064 -1.5393 2.9626 CI includes 0, so no evidence of mediation

Partially standardized indirect effect(s) of X on Y:

Effect BootSE BootLLCI BootULCI

Delt_TMT .0642 .1062 -.1519 .2875
